# IL-10-producing innate lymphoid cells restrain γδT17-driven psoriatic inflammation

**DOI:** 10.1016/j.isci.2026.117026

**Published:** 2026-07-31

**Authors:** Min Geun Jo, Min Yeong Choi, Keun Young Min, Jeong Won Park, Juhyun Shin, Ji-Ae Lee, Sunsook Hwang, Geunwoong Noh, Young Mi Kim, Wahn Soo Choi, Hyuk Soon Kim

**Affiliations:** 1 Department of Immunology, School of Medicine, Konkuk University, Chungju 27478, Korea; 2 Department of Microbiology and Immunology, Weill Cornell Medicine, New York, NY 10021, USA; 3 Department of Biomedical Sciences, College of Natural Science and Department of Health Sciences, The Graduate School of Dong-A University, Busan 49315, Korea; 4 Department of Allergy, Allergy and Clinical Immunology Center, Cheju Halla General Hospital, Jeju 63127, Korea; 5 Department of Preventive Pharmacy, College of Pharmacy, Duksung Women’s University, Seoul 01369, Korea

**Keywords:** regulatory ILCs, γδ T cells, IL-10, psoriasis, CCR2

## Abstract

Psoriasis is a chronic inflammatory dermatosis driven by the interleukin (IL)-23-IL-17 axis, with γδ T cells as key IL-17A producers in lesional skin. IL-10 counter-regulates this pathway, and innate lymphoid cells (ILCs) are emerging tissue-resident immune regulators. Here we show that imiquimod (IMQ)-induced psoriatic inflammation elicits a programmed death-ligand 1 (PD-L1)^hi^ stem cell antigen-1 (Sca-1)^+^ ILC2-like subset enriched for IL-10 (ILC2_10_) that acts as an innate brake on the γδ T17 axis. Adoptive transfer of ILC2_10_ reduced clinical severity and epidermal hyperplasia and suppressed γδ T cell IL-17A production and CCR2 expression in coculture; *Il10*^−/−^-derived ILC2_10_ lacked these effects, indicating that IL-10 is required. Reanalysis of human psoriasis single-cell RNA sequencing datasets identified a PD-L1^+^IL-10^+^ ILC population in lesional skin. These findings define ILC2_10_ as an IL-10-dependent innate regulatory checkpoint restraining γδ T17-driven psoriatic inflammation, revealing a previously unrecognized innate regulatory layer in this disease.

## Highlights

• PD-L1^high^ IL-10-enriched ILC2-like cells expand in IMQ-induced lesional skin
• Adoptive transfer of ILC2_10_ cells suppresses psoriatic skin inflammation
• IL-10 is required for suppression of γδ T cell IL-17A and CCR2 expression
• Human psoriatic lesions contain PD-L1^+^IL-10^+^ innate lymphoid cells

## Graphical abstract

**Figure:**
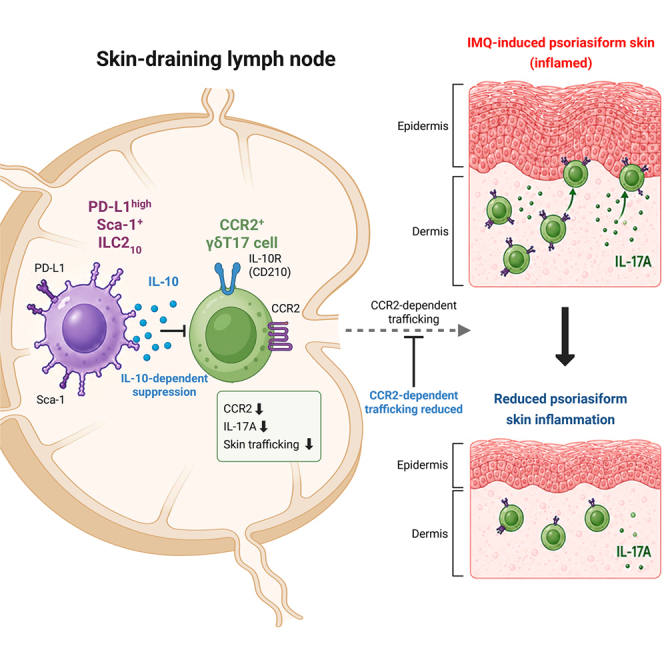

## Teaser

Dermatology; Immunology; Cell biology

## Introduction

Psoriasis is a chronic inflammatory disorder of the skin characterized by excessive proliferation of keratinocytes and dense leukocyte infiltration, largely driven by the interleukin (IL)-23–IL-17 pathway.^1^^,^^2^^,^^3^ Antibodies targeting IL-17 or IL-23 have improved disease control, yet many patients exhibit only partial responses, experience secondary loss of efficacy, or relapse after treatment is discontinued.^4^^,^^5^^,^^6^ These limitations underscore the need to define lesion-localized regulatory mechanisms that may act in concert with cytokine blockade to restrain the pathogenesis of the IL-23–IL-17 axis.^1^^,^^2^^,^^3^

Among the effector populations that initiate and maintain psoriatic inflammation, IL-17-producing γδ T cells (γδ T17 cells) are key contributors to lesion development and spread in the imiquimod (IMQ)-induced mouse model.^7^^,^^8^^,^^9^ Under inflammatory conditions, γδ T17 cells adopt a C-C chemokine receptor (CCR2)-centered migratory program that promotes their recruitment into lesional skin and is associated with local tissue damage.^10^^,^^11^^,^^12^ However, the mechanisms by which innate regulatory pathways restrain this γδ T17–CCR2 pathogenic axis remain poorly defined.

IL-10 is an anti-inflammatory cytokine whose expression is decreased in psoriatic plaques and restored with effective therapy, which is consistent with its role in limiting cutaneous inflammation.^13^^,^^14^ In skin inflammation models such as contact hypersensitivity (CHS), we and others have described a type 2 innate lymphoid cell (ILC2)-derived regulatory subset with high IL-10 expression (hereafter termed ILC2_10_ cells) that limits tissue inflammation.^15^ Sca-1 has been widely used as a murine ILC2-associated marker,^16^ and PD-L1 expression has been reported on activated ILC2s and on IL-10-producing regulatory ILC2-like populations.^17^ In our previous CHS study,^15^ PD-L1^hi^Sca-1^+^ ILCs were markedly enriched for IL-10 expression and showed predominant GATA3 expression with increased *Gata3* mRNA, consistent with an ILC2-like regulatory phenotype. Based on these observations, we used the PD-L1^hi^Sca-1^+^ phenotype not as a *de novo* lineage definition, but as a surface phenotype to enrich and track a previously validated regulatory ILC2-like subset in the IMQ model. Whether a comparable IL-10-producing ILC subset is induced and maintained in IL-23/IL-17-driven IMQ-induced psoriatic inflammation remains unknown. It is also unclear whether such cells modulate the γδ T17–CCR2 axis, including CCR2 programming and the recruitment of γδ T17 cells into lesional skin.

In this study, we used the IMQ-induced psoriatic skin inflammation model to characterize lesion-specific changes in programmed death-ligand 1 (PD-L1)^hi^ stem cell antigen-1 (Sca-1)^+^ ILC2_10_ cells and investigated whether they regulate γδ T17-cell CCR2 programming and recruitment into lesional skin in an IL-10-dependent manner. In parallel, we reanalyzed independent human psoriasis skin single-cell RNA sequencing cohorts to assess the presence and distribution of IL-10^+^PD-L1^+^ ILCs in lesional skin and to evaluate whether key features of the regulatory axis observed in mice are also seen in human psoriasis.

## Results

### IL-10^+^ ILCs expand and correlate with γδ T cell accumulation in IMQ-induced lesions

**Figure 1:**
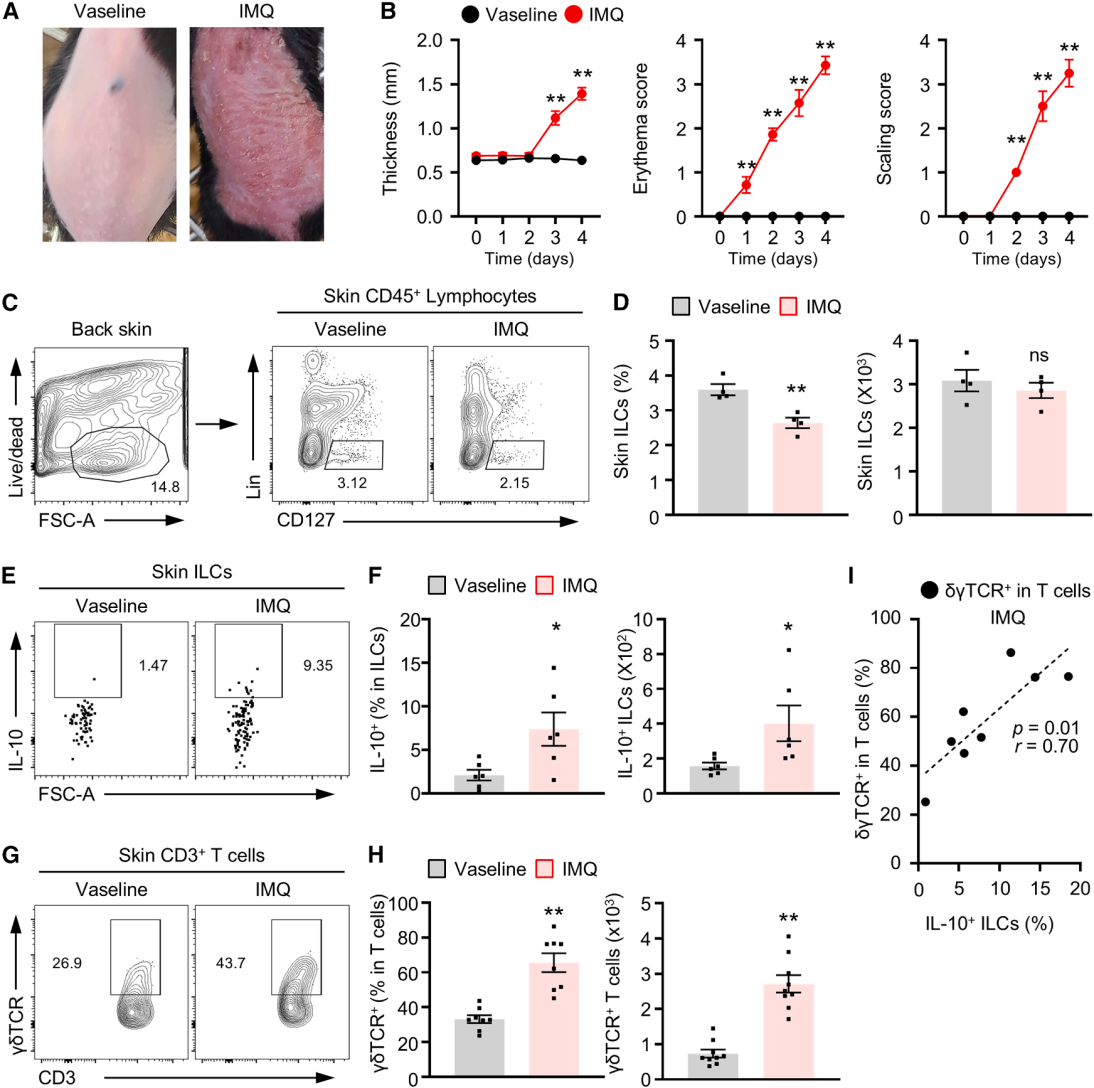
IMQ-induced psoriatic inflammation is associated with expansion of IL-10^+^ innate lymphoid cells and accumulation of γδ T cells in the skin (A) Representative dorsal skin images of mice treated daily for 4 days with Vaseline or 5% IMQ. (B) PASI scoring curves quantifying erythema, scaling, and thickness in Vaseline- and IMQ-treated mice (*n* = 6). (C) Flow cytometric gating strategy for Lin^−^CD127^+^ ILCs among live lymphocytes in the back skin of Vaseline- and IMQ-treated mice. (D) Bar graphs show the percentage (left) and absolute number (right) of skin Lin^−^CD127^+^ ILCs in Vaseline- and IMQ-treated mice; IMQ treatment reduces ILC frequency without significantly altering total counts (*n* = 4). (E) Representative plots of IL-10^+^ ILCs in skin from Vaseline- and IMQ-treated mice. (F) Bar graphs showing the percentage (left) and absolute number (right) of skin IL-10^+^ ILCs in Vaseline-versus IMQ-treated mice (*n* = 6). (G) Representative plots of γδTCR^+^ T cells in skin from Vaseline- and IMQ-treated mice. (H) Bar graphs showing the percentage (left) and absolute number (right) of skin γδTCR^+^ T cells in Vaseline-versus IMQ-treated mice (*n* = 6). (I) Scatterplot showing the correlation between the frequency of skin γδTCR^+^ T cells and IL-10^+^ ILC counts across individual mice (*n* = 8). Data are presented as mean ± SEM. Statistical significance is indicated as ∗*p* < 0.05, ∗∗*p* < 0.01, and ns for not significant. Statistical analyses were performed as follows: PASI time-course curves in (B) were analyzed using two-way repeated-measures ANOVA with Sidak’s multiple comparisons test comparing Vaseline and IMQ at each day. Comparisons between Vaseline and IMQ in (D, F, and H) were analyzed using two-sided unpaired Student’s t-tests. The correlation in (I) was assessed by Pearson’s correlation.

To determine how the ILC compartment changes during IMQ-induced psoriatic inflammation, we induced psoriasis-like skin inflammation by applying 5% IMQ cream to the back skin once daily for 4 days (Figure S1A). IMQ treatment induced substantial epidermal thickening and psoriasiform changes (Figures 1A and 1B), which is consistent with the robust induction of psoriasis-like inflammation.^18^ Flow cytometric analysis of the back skin revealed that the proportion of total Lin^−^CD127^+^ ILCs among leukocytes decreased, while their absolute counts did not significantly change (Figures 1C and 1D). In contrast, the percentage of IL-10-producing ILCs (IL-10^+^Lin^−^CD127^+^) increased, with both the frequency and the number of IL-10^+^ ILCs increasing in lesional skin after IMQ treatment (Figures 1E and 1F). In parallel, γδ T cell receptor (γδTCR)^+^ T cells accumulated in lesional skin (Figures 1G and 1H), whereas other major IL-10–producing populations, including IL-10^+^ regulatory T cells (Tregs) and IL-10^+^ macrophages, were reduced (Figure S1B). On a per-mouse basis, the proportion of IL-10^+^ ILCs correlated strongly with the abundance of γδTCR^+^ T cells (r = 0.70, *p* = 0.01; *n* = 8; Figure 1I). Thus, IMQ-induced psoriatic inflammation is accompanied by a selective expansion of IL-10^+^ ILCs that closely tracks with γδ T cell accumulation in lesional skin, prompting us to characterize the phenotype and function of this expanded IL-10^+^ ILC population.

### PD-L1^hi^Sca-1^+^ ILC2_10_ expands and represents a major IL-10^+^ ILC subset in IMQ-induced psoriatic inflammation

**Figure 2:**
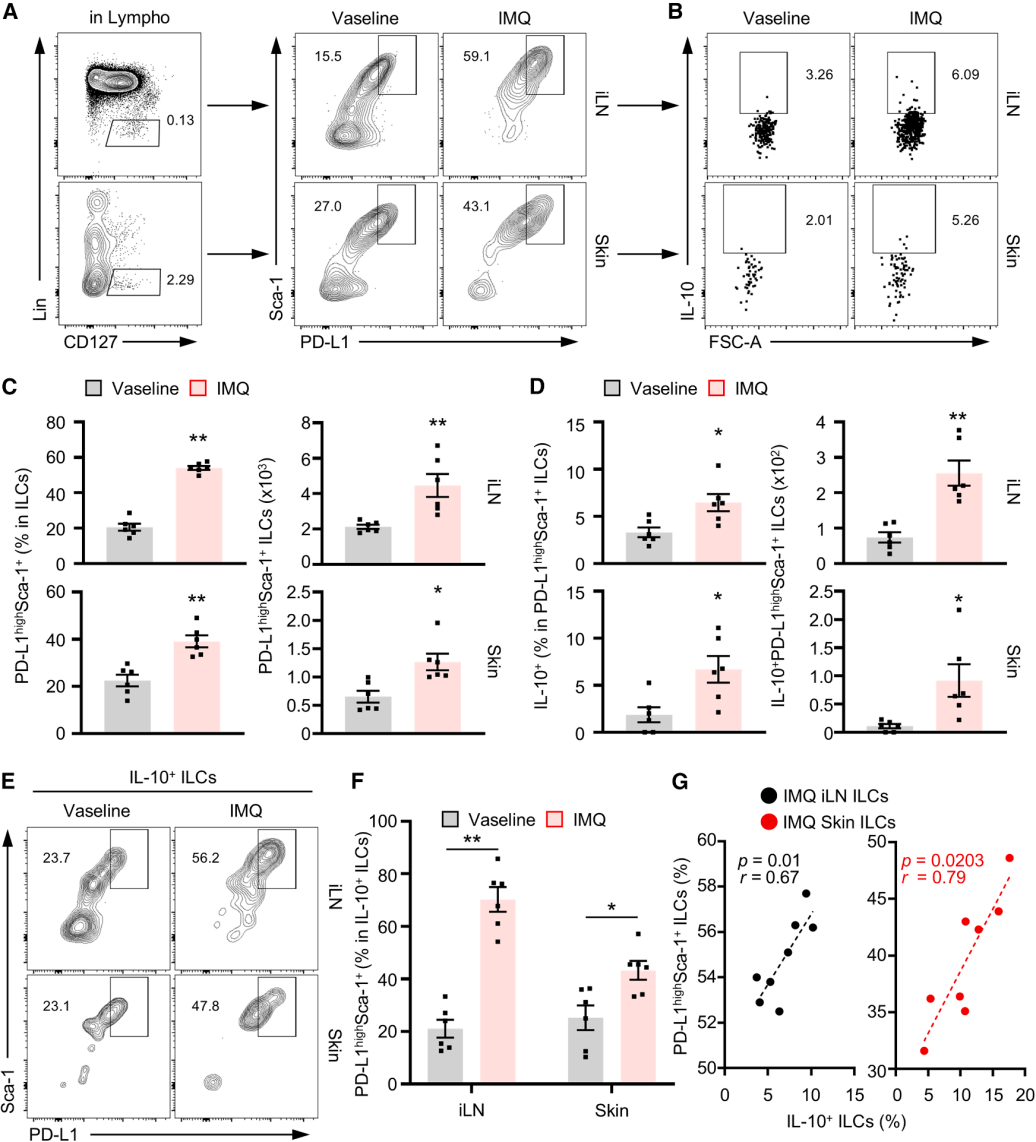
IMQ-induced psoriatic inflammation selectively expands PD-L1^hi^Sca-1^+^ ILC2_10_ within the IL-10^+^ ILC compartment (A) Flow cytometric gating of PD-L1^hi^Sca-1^+^ cells gated on Lin^−^CD127^+^ ILCs in the iLN (top) and skin (bottom) from Vaseline- and IMQ-treated mice. (B) Representative plots show IL-10 expression within the PD-L1^hi^Sca-1^+^ ILC subset in iLN (top) and skin (bottom) of Vaseline- and IMQ-treated mice. (C) Bar graphs of PD-L1^hi^Sca-1^+^ ILCs in iLN (top) and skin (bottom): frequency (percentage of total ILCs; left) and absolute counts (right); IMQ treatment significantly increases both measures (*n* = 6). (D) Bar graphs of IL-10^+^ cells within the PD-L1^hi^Sca-1^+^ ILC compartment in Vaseline-versus IMQ-treated mice, shown separately for iLN (top) and skin (bottom): percentage of IL-10^+^ cells among PD-L1^hi^Sca-1^+^ ILCs (left) and absolute cell counts (right); IMQ treatment increases both the frequency and number of IL-10-producing PD-L1^hi^Sca-1^+^ ILCs (*n* = 6). (E) Representative plots of PD-L1 versus Sca-1 expression gated on IL-10^+^ ILCs in iLN (top) and skin (bottom) of Vaseline- and IMQ-treated mice. (F) Bar graphs of PD-L1^hi^Sca-1^+^ frequency among IL-10^+^ ILCs in iLN (left) and skin (right); the PD-L1^hi^Sca-1^+^ subset is preferentially expanded following IMQ treatment (*n* = 6). (G) Scatterplots showing the correlation between IL-10^+^ ILC frequency and PD-L1^hi^Sca-1^+^ subset frequency in iLN (black) and skin (red) across individual mice (*n* = 8). Data are presented as mean ± SEM. Statistical significance is indicated as ∗*p* < 0.05, ∗∗*p* < 0.01, and ns for not significant. Comparisons between Vaseline and IMQ in (C, D, and F) were analyzed using two-sided unpaired Student’s t-tests. Correlations in (G) were assessed by Pearson’s correlation analysis.

We next characterized the phenotype of IL-10-producing ILCs in psoriatic inflammation by evaluating PD-L1 and Sca-1 expression on Lin^−^CD127^+^ ILCs from skin and draining inguinal lymph nodes (iLNs) (Figure 2A). IMQ treatment led to the expansion of PD-L1^hi^Sca-1^+^ ILCs (hereafter ILC2_10_ cells), with both their frequencies and absolute numbers increasing in iLNs and lesional skin compared with those in Vaseline-treated controls (Figures 2A–2D). Consistent with the data shown in Figure 1, ILC2_10_ cells accounted for a greater fraction of the IL-10^+^ ILC pool in the IMQ-treated mice than in the Vaseline control mice, reaching approximately half of the number of IL-10^+^ ILCs in both the iLNs and skin (Figures 2E and 2F). Across individual IMQ-treated mice (*n* = 8), the proportion of ILC2_10_ cells correlated positively with the overall frequency of IL-10^+^ ILCs (iLN: r = 0.67, *p* = 0.01; skin: r = 0.79, *p* = 0.02; Figure 2G). Thus, in IMQ-induced psoriatic inflammation, the ILC2_10_ population expands in parallel with IL-10^+^ ILCs and emerges as a major subset within the IL-10-producing ILC compartment.

### Lesional human psoriatic skin contains increased proportions of PD-L1^+^, IL-10^+^, and PD-L1^+^IL-10^+^ ILCs

**Figure 3:**
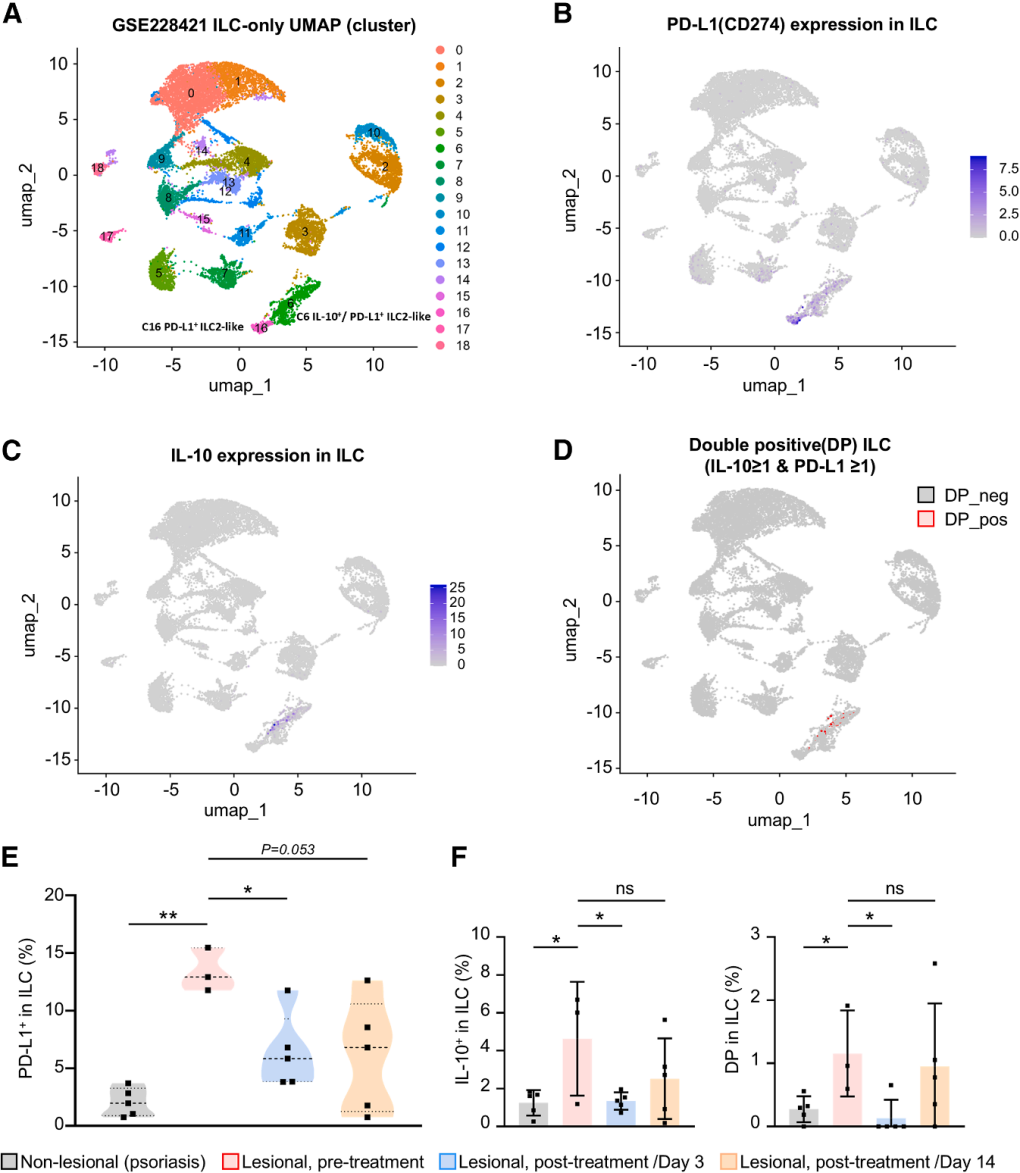
Lesional human psoriatic skin is enriched for PD-L1^+^ and PD-L1^+^IL-10^+^ ILCs (A) UMAP of ILCs from the GSE228421 human psoriasis skin scRNA-seq dataset, colored by transcriptional cluster and annotated to highlight marker-relevant ILC2-like clusters 6 and 16. Cluster 6 is annotated as an IL-10^+^/PD-L1(CD274)^+^ double-positive (DP) ILC2-like cluster containing most IL10-expressing and PD-L1^+^IL-10^+^ double-positive ILCs, whereas cluster 16 is annotated as a PD-L1^+^ ILC2-like cluster showing prominent PD-L1 expression. (B) Feature plot shows PD-L1 (CD274) expression across ILC clusters. (C) Feature plot shows IL-10 expression, which is rare and largely confined to cluster 6. (D) UMAP highlighting PD-L1^+^IL-10^+^ double-positive (DP) ILCs (IL10 ≥ 1 and CD274 ≥ 1 UMI; red) overlaid on all remaining ILCs (gray). (E) Violin/dot plots show the percentage of PD-L1^+^ ILCs among total ILCs in non-lesional psoriatic skin (gray), lesional pre-treatment (V1_L, red), lesional post-treatment Day 3 (V2_L, blue), and lesional post-treatment Day 14 (V3_L, green). Each dot represents one sample (V1_L, *n* = 3; V1_NL, V2_L, and V3_L, *n* = 5); PD-L1^+^ ILCs are significantly increased in pre-treatment lesional skin compared with non-lesional and Day 3 lesional samples. (F) Bar/dot plots show the frequencies of IL-10^+^ ILCs (left) and PD-L1^+^IL-10^+^ DP ILCs (right) among total ILCs across the same four groups; despite their overall rarity, PD-L1^+^IL-10^+^ ILCs are mainly observed in pre-treatment lesional skin. Data are presented as mean ± SEM (E and F). Statistical significance in (E and F) was analyzed using one-way ANOVA followed by Tukey’s multiple comparisons test; ∗*p* < 0.05; ns, not significant.

Building on these murine findings, we examined the distribution of PD-L1^+^IL-10^+^ ILCs in human psoriatic skin by reanalyzing the ILC compartment in the GSE228421 human psoriasis skin single-cell RNA-seq dataset (Figures 3A–3D).^19^ UMAP clustering of the ILC compartment resolved multiple transcriptionally distinct subclusters (Figure 3A). PD-L1 (CD274) was expressed across several clusters but was most prominent in clusters 16 and 6 (Figure 3B). IL-10 transcripts were detected at low frequency and were largely confined to cluster 6 (Figure 3C). PD-L1^+^IL-10^+^ ILCs were similarly restricted, with most double-positive cells mapping to cluster 6 (Figure 3D). Consistent with the subtype-like annotation described later in discussion and in Figure S2A, clusters 6 and 16 were annotated in the revised UMAP as marker-relevant ILC2-like clusters.

In this cohort, each patient contributed a pretreatment lesional (V1_L) and nonlesional (V1_NL) sample, as well as posttreatment lesional biopsies on day 3 (V2_L) and day 14 (V3_L). For the present analysis, we applied a predefined QC threshold (nILCs ≥100) and included only samples with sufficient ILC numbers, which led to the exclusion of some lesional samples owing to low ILC counts. As a result, 3 V1_L samples and 5 samples each for V1_NL, V2_L, and V3_L were retained for quantitative analysis (Figures 3E and 3F). Statistical comparisons were performed by treating the QC-passing samples as independent (unpaired) groups.

Quantitatively, the proportion of PD-L1^+^ ILCs was greater in pretreated lesional skin (V1_L) than in nonlesional skin (V1_NL), and the proportion of PD-L1^+^ ILCs remained higher in V1_L samples than in day 3 lesional samples (V2_L). Compared with the population in V3_L samples, that in V2_L samples tended to decrease (*p* = 0.053), which was consistent with a decrease followed by partial recovery over the treatment course, although this did not reach conventional statistical significance (Figure 3E).

The frequency of IL-10^+^ ILCs was also greater in V1_L than in both V1_NL and V2_L samples, whereas no significant difference was observed between V1_L and V3_L samples (Figure 3F, left). PD-L1^+^IL-10^+^ ILCs (double-positive (DP)), while overall a minor population, were detected at higher proportions in pretreated lesional skin (V1_L) than in V1_NL and V2_L samples, while the levels in V1_L and V3_L samples did not significantly differ (Figure 3F, right).

Taken together, these data indicate that PD-L1^+^ ILCs, IL-10^+^ ILCs, and PD-L1^+^IL-10^+^ ILCs are more abundant in pretreated lesional skin than in nonlesional skin and lesions in early treatment and that PD-L1^+^IL-10^+^ ILCs, despite their low frequency, have a lesion-biased distribution in human psoriasis. To further define the identity of these human ILCs, we performed subtype-like annotation using *GATA3*, *IL1RL1* (ST2), *KLRG1*, *HPGDS*, *PTGDR2*, and *IL7R* for ILC2-like cells, *RORC* for ILC3-like cells, and *TBX21* for ILC1-like cells. This analysis showed that the predominant fraction of human skin ILCs displayed an ILC2-like signature, and this tendency was further enriched among PD-L1^+^ ILCs and PD-L1^+^IL-10^+^ ILCs (Figure S2A). We additionally performed a transcript-based analysis of γδ T cells in the same dataset, defining γδ T cells as *TRDC* > 0, *TRGC1*/*2* > 0, and *TRAC* = = 0. In V1 paired samples, after exclusion of one non-lesional sample with an extreme value that disproportionately influenced the paired comparison, γδ T cell proportions were higher in lesional skin in three of four patients, whereas one patient showed a higher value in non-lesional skin. Because γδ T cell counts were sparse overall and the analysis remained sensitive to sample-level variability, we interpret this analysis as exploratory (Figure S2B).

### Adoptive transfer of PD-L1^hi^Sca-1^+^ ILC2_10_ cells attenuates IMQ-induced psoriatic inflammation and dampens lesional γδ T17-cell responses

**Figure 4:**
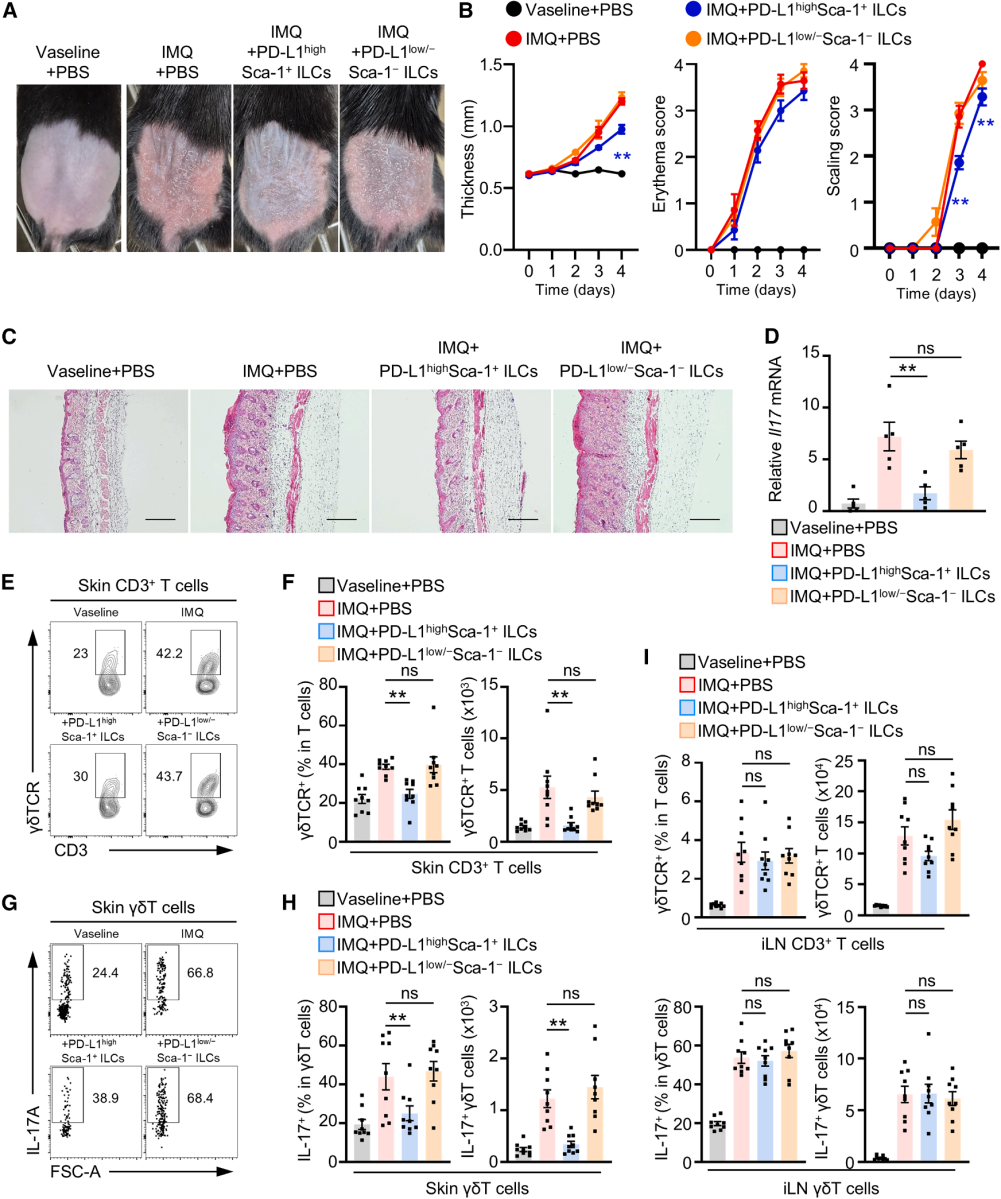
Adoptive transfer of PD-L1^hi^Sca-1^+^ ILCs suppresses γδ T cell-driven psoriatic inflammation (A) Representative dorsal skin images of wild-type mice treated daily for 4 days with Vaseline, IMQ+PBS, IMQ+PD-L1^hi^Sca-1^+^ ILCs, or IMQ+PD-L1^lo/−^Sca-1^-^ ILCs. (B) Bar graph of PASI scores quantifying erythema, scaling, and thickness across treatment groups; transfer of PD-L1^hi^Sca-1^+^ ILCs significantly reduces clinical severity (*n* = 6). (C) Representative H&E-stained back skin sections show that IMQ+PBS control mice developed marked epidermal acanthosis/hyperplasia with increased inflammatory infiltrates in lesional skin, whereas these histologic changes were attenuated in mice receiving PD-L1^hi^Sca-1^+^ ILC2_10_ cells but not in mice receiving PD-L1^lo/−^Sca-1^-^ ILCs. (D) qPCR analysis of skin *Il17a* mRNA levels normalized to β-actin; *Il17a* expression is significantly decreased in the IMQ+ PD-L1^hi^Sca-1^+^ ILC group compared with IMQ+PBS (*n* = 5). Representative flow cytometry plots (E) and corresponding bar graphs (F) showing the frequency (left) and absolute counts (right) of γδTCR^+^ T cells among CD45^+^ lymphocytes in back skin; PD-L1^hi^Sca-1^+^ ILC transfer reduces γδTCR^+^ T cell infiltration (*n* = 9). Representative FACS plots (G) and corresponding bar graphs (H) showing the frequency (left) and absolute counts (right) of IL-17A^+^ cells within the skin γδTCR^+^ compartment; PD-L1^hi^Sca-1^+^ ILC transfer suppresses effector cytokine production (*n* = 9). (I) Bar graphs of the frequency and absolute counts of γδTCR^+^ and IL-17A^+^ γδ T cells in iLN across treatment groups; no significant changes are observed (*n* = 9). Data are presented as mean ± SEM. Statistical significance is indicated as ∗*p* < 0.05, ∗∗*p* < 0.01; ns, not significant. PASI time-course curves in (B) were analyzed using two-way repeated-measures ANOVA with factors of treatment and time, followed by Sidak’s multiple comparisons test between treatment groups at each day. Group comparisons in (D, F, H, and I) were analyzed using one-way ANOVA followed by Tukey’s multiple comparisons test. Scale bars, 100 μm.

To test the protective role of ILC2_10_ cells in IMQ-induced psoriatic inflammation, we used an adoptive transfer approach. WT donor mice were administered IL-33 to expand splenic ILCs,^20^^,^^21^^,^^22^ after which PD-L1^hi^Sca-1^+^ ILC2_10_ cells and PD-L1^lo/−^Sca-1^-^ ILCs (non-ILC2_10_ cells) were sorted from the spleen, and each population was transferred intravenously into separate recipient mice 1 day before IMQ application (Figures S3A–S3C and S4A). IL-33 treatment increased both the total number of splenic ILCs and the PD-L1^hi^Sca-1^+^ ILC2_10_ subset, and postsort analyses confirmed purities greater than 95% for the ILC2_10_ and non-ILC2_10_ fractions (Figures S3B and S3C). Compared with IMQ+PBS (phosphate-buffered saline) control mice, mice receiving ILC2_10_ cells showed smaller increases in lesional skin thickness and scaling scores, and histologic analysis revealed attenuated epidermal acanthosis/hyperplasia with reduced inflammatory infiltrates in lesional skin (Figures 4A–4C). In contrast, the transfer of non-ILC2_10_ cells did not result in comparable clinical or histologic improvement (Figures 4A–4C).

At the molecular level, the abundance of the *Il17a* transcript in lesional skin was lower in ILC2_10_ recipients than in IMQ+PBS controls (Figure 4D). Flow cytometric analysis further revealed that the frequency and absolute number of γδTCR^+^ T cells were reduced in the lesional skin of mice receiving ILC2_10_ cells. In addition, the frequencies and absolute counts of IL-17A^+^ cells within the γδ T cell compartment decreased, which was consistent with the decreased γδ T17-cell response (Figures 4E–4H). These changes were not observed in iLNs (Figure 4I), indicating that the regulatory effect of ILC2_10_ cells was most evident in the skin compartment. In contrast, conventional Th17 (CD4^+^IL-17A^+^) cells in lesional skin were not significantly altered by ILC2_10_-cell transfer (Figures S4B–S4E). Taken together, these findings suggest that the adoptive transfer of ILC2_10_ cells ameliorated IMQ-induced psoriatic inflammation, which was accompanied by clinical improvement, attenuation of histologic inflammatory changes in lesional skin, and reduced γδ T17-cell readouts. To assess whether the regulatory influence of ILC2_10_ cells extends beyond the γδ T17-cell response, we next examined Foxp3^+^CD4^+^ Tregs among skin CD3^+^ T cells under the same experimental conditions. Using Foxp3-based Treg gating, neither the frequency nor the absolute number of Foxp3^+^CD4^+^ Tregs differed between IMQ+PBS controls and ILC2_10_-cell recipients (Figures S5A and S5B). In the macrophage compartment, however, the fraction of CD206^+^ subsets, which was reduced by IMQ treatment, was greater in mice receiving ILC2_10_ cells than in IMQ+PBS control mice in both iLNs and lesional skin, and in iLNs, the absolute number of CD206^+^ macrophages partially recovered (Figures S5C and S5D). Consistently, qPCR analysis of lesional skin revealed increased *Arg1* expression and decreased *Nos2* expression in mice receiving ILC2_10_ cells (Figure S5E). These findings suggest that the weakening of γδ T17-cell readouts after ILC2_10_-cell transfer is accompanied by M2-like macrophage reprogramming.^23^^,^^24^^,^^25^ Whether this macrophage phenotype reflects a direct effect of ILC2_10_ cells or arises secondarily from a reduced inflammatory burden cannot be distinguished from the present data and will require further mechanistic studies.

### IL-10 is essential for the regulatory function of PD-L1^hi^Sca-1^+^ ILCs in psoriatic inflammation

**Figure 5:**
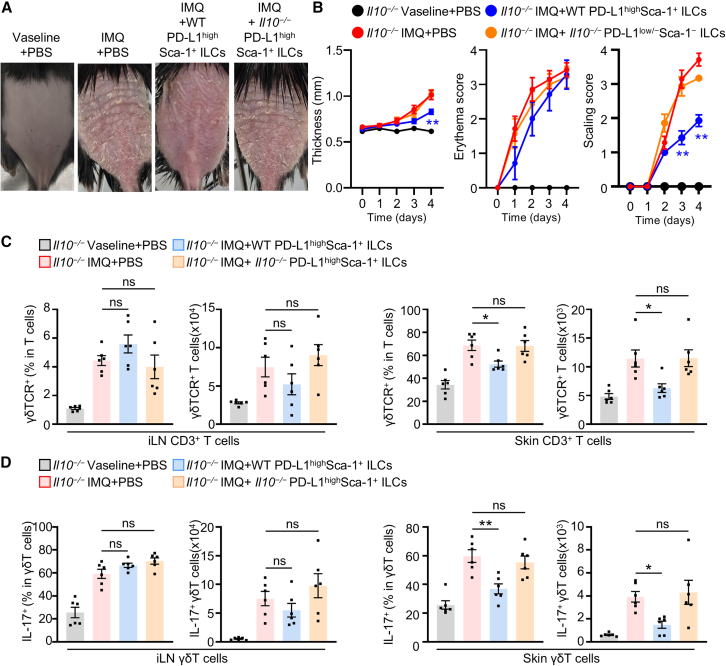
IL-10 is essential for PD-L1^hi^Sca-1^+^ ILC-mediated suppression of psoriatic inflammation (A) Representative dorsal skin images of *Il10*^−/−^ mice treated daily for 4 days with Vaseline, IMQ+PBS, IMQ+WT PD-L1^hi^Sca-1^+^ ILCs, or IMQ+*Il10*^−/−^ PD-L1^hi^Sca-1^+^ ILCs. (B) Bar graphs of PASI scores quantifying erythema, scaling, and thickness across treatment groups; only transfer of WT PD-L1^hi^Sca-1^+^ ILCs significantly ameliorates clinical disease (*n* = 6). (C) Bar graphs of γδTCR^+^ T cells among CD45^+^ lymphocytes in iLN (left two panels) and skin (right two panels): frequency (top panels) and absolute counts (bottom panels); skin γδTCR^+^ T cells are reduced only in mice receiving WT PD-L1^hi^Sca-1^+^ ILCs (*n* = 6). (D) Bar graphs of IL-17A^+^ γδ T cells in iLN (left two panels) and skin (right two panels): frequency (top panels) and absolute counts (bottom panels); reduction of IL-17A^+^ γδ T cells is observed only in the skin of mice receiving WT PD-L1^hi^Sca-1^+^ ILCs (*n* = 6). Data are presented as mean ± standard error of the mean (SEM). Statistical significance is indicated as ∗*p* < 0.05, ∗∗*p* < 0.01; ns, not significant. PASI time-course curves in (B) were analyzed using two-way repeated-measures ANOVA with Sidak’s multiple comparisons test between treatment groups at each day. Group comparisons in (C and D) were analyzed using one-way ANOVA followed by Tukey’s multiple comparisons test.

To test whether IL-10 is required for the anti-inflammatory activity of ILC2_10_ cells, we sorted equal numbers of WT-derived ILC2_10_ cells and *Il10*^−/−^ ILC2_10_ cells from donor mice and intravenously transferred each population into *Il10*^−/−^ recipient mice 1 day before IMQ application (Figures 5A and 5B). In recipients of WT ILC2_10_ cells, the IMQ-induced increase in lesional skin thickness and scaling was significantly attenuated. In contrast, mice receiving *Il10*^−/−^ ILC2_10_ cells developed lesions comparable to those of the IMQ+PBS control mice, without detectable protection (Figures 5A and 5B). These data indicate that the *in vivo* protective effect of ILC2_10_ cells requires IL-10.

To determine whether this clinical divergence was linked to the suppression of the γδ T17-cell response, we next analyzed γδ T cell readouts. In the lesional skin of WT ILC2_10_-cell recipients, both the frequency and absolute number of γδTCR^+^ T cells were significantly reduced, and the frequency and absolute number of γδ T17 cells were also decreased. These changes were not detected in *Il10*^−/−^ ILC2_10_-cell recipients (Figures 5C and 5D). In draining iLNs, the γδ T cell and γδ T17-cell parameters were not significantly altered across the groups, which is consistent with the effect being most evident in lesional skin (Figures 5C and 5D). Overall, in the IMQ-induced psoriatic model, ILC2_10_ cell-mediated protection was associated with IL-10-dependent attenuation of γδ T17-cell readouts in the skin.

### PD-L1^hi^Sca-1^+^ ILCs suppress CCR2 expression in γδ T cells in an IL-10-dependent manner

**Figure 6:**
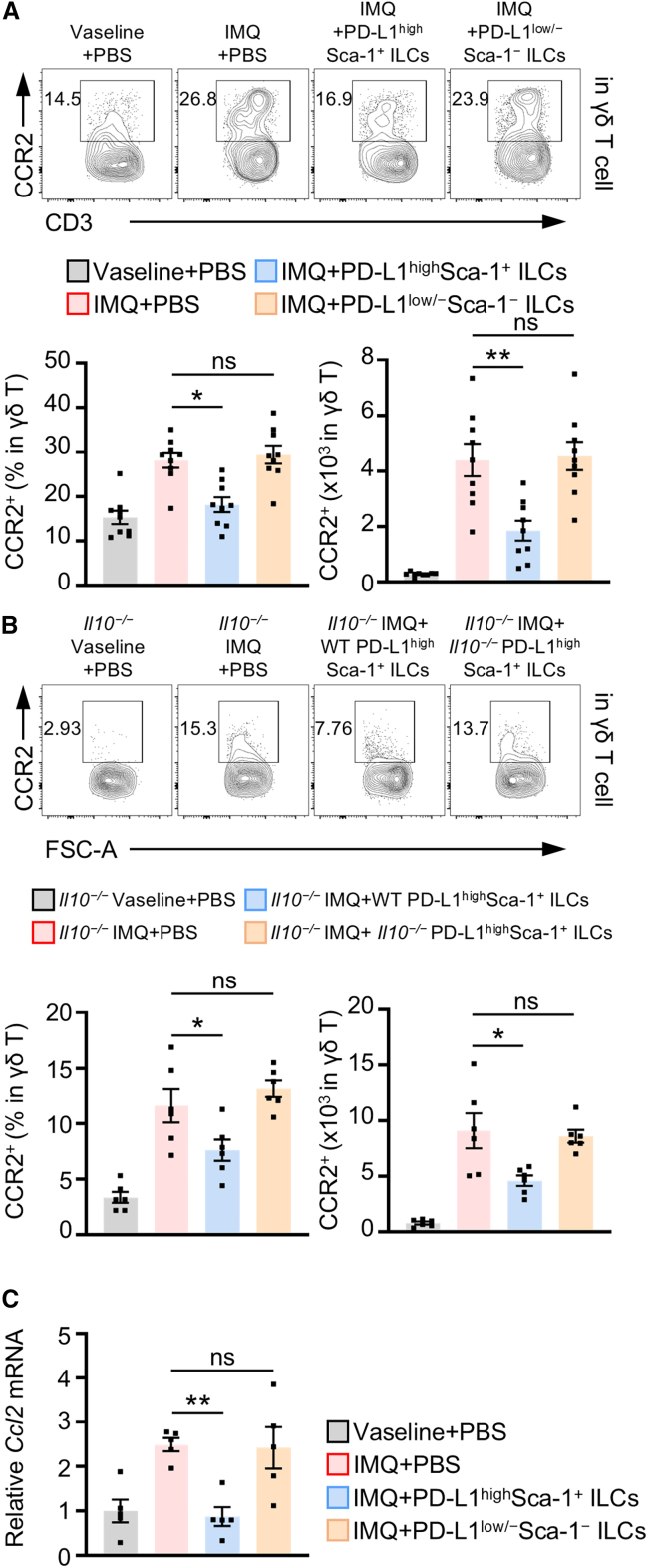
PD-L1^hi^Sca-1^+^ ILCs suppress CCR2 expression in γδ T cells in an IL-10-dependent manner and concomitantly reduce CCL2 levels in psoriatic skin (A) Representative flow cytometry plots of CCR2 expression on γδTCR^+^ T cells in iLN from wild-type mice treated with Vaseline+PBS, IMQ+PBS, IMQ+PD-L1^hi^Sca-1^+^ ILCs, or IMQ+PD-L1^lo/−^Sca-1^-^ ILCs. Bar graphs show the percentage of CCR2^+^ γδTCR^+^ cells (left) and absolute CCR2^+^ cell counts (right) in each group; transfer of PD-L1^hi^Sca-1^+^ ILCs significantly reduces CCR2 expression (*n* = 9). (B) Representative flow cytometry plots of CCR2 on skin γδTCR^+^ T cells from *Il10*^−/−^ mice treated with Vaseline+PBS, IMQ+PBS, IMQ+WT PD-L1^hi^Sca-1^+^ ILCs, or IMQ+*Il10*^−/−^ PD-L1^hi^Sca-1^+^ ILCs. Bar graphs of CCR2^+^ frequency (left) and absolute counts (right); suppression of CCR2 is observed only in mice receiving WT PD-L1^hi^Sca-1^+^ ILCs, indicating that this effect requires IL-10 from the transferred ILCs (*n* = 6). (C) qPCR analysis of skin *Ccl2* mRNA (normalized to β-actin) in wild-type mice treated as in (A); only transfer of PD-L1^hi^Sca-1^+^ ILCs significantly lowers *Ccl2* expression (*n* = 5). Data are presented as mean ± SEM. Statistical significance is indicated as ∗*p* < 0.05, ∗∗*p* < 0.01; ns, not significant. Group comparisons in (A, B and C) were analyzed using one-way ANOVA followed by Tukey’s multiple comparisons test.

Because ILC2_10_-cell transfer most clearly affected lesional skin, we evaluated the expression of CCR2 on γδ T cells as a marker of their tissue-homing ability. IMQ treatment increased both the frequency and absolute number of CCR2^+^ γδ T cells in draining iLNs; notably, compared with those in IMQ+PBS control mice, both measures were significantly reduced in ILC2_10_ cell-treated mice (Figure 6A), whereas no reduction in CCR2 expression was observed in recipients of non-ILC2_10_ cells (Figure 6A). The overall γδ T cell pool and IL-17 responses in iLNs were not altered (Figure 4I).

To test the degree of IL-10 dependence, equal numbers of WT ILC2_10_ or *Il10*^−/−^ ILC2_10_ cells were transferred into *Il10*^−/−^ recipient mice, and the expression of CCR2 was compared. Only recipients of WT ILC2_10_ cells showed significant reductions in the frequency and absolute number of CCR2^+^ γδ T cells in lesional skin, whereas *Il10*^−/−^ ILC2_10_ cells did not elicit these changes (Figure 6B). These data indicate that CCR2 modulation by ILC2_10_ cells is IL-10 dependent.

We next quantified chemotactic signaling in skin by measuring *Ccl2* mRNA levels. IMQ-induced *Ccl2* expression was significantly lower in WT ILC2_10_-cell recipients but not in non-ILC2_10_-cell recipients (Figure 6C), which is consistent with a parallel decrease in the local CC Motif Chemokine Ligand 2 (CCL2) milieu accompanied by CCR2 attenuation.

### IL-10-dependent suppression of IL-17A production and CCR2 expression in γδ T cells by PD-L1^hi^Sca-1^+^ ILC2_10_ in coculture

**Figure 7:**
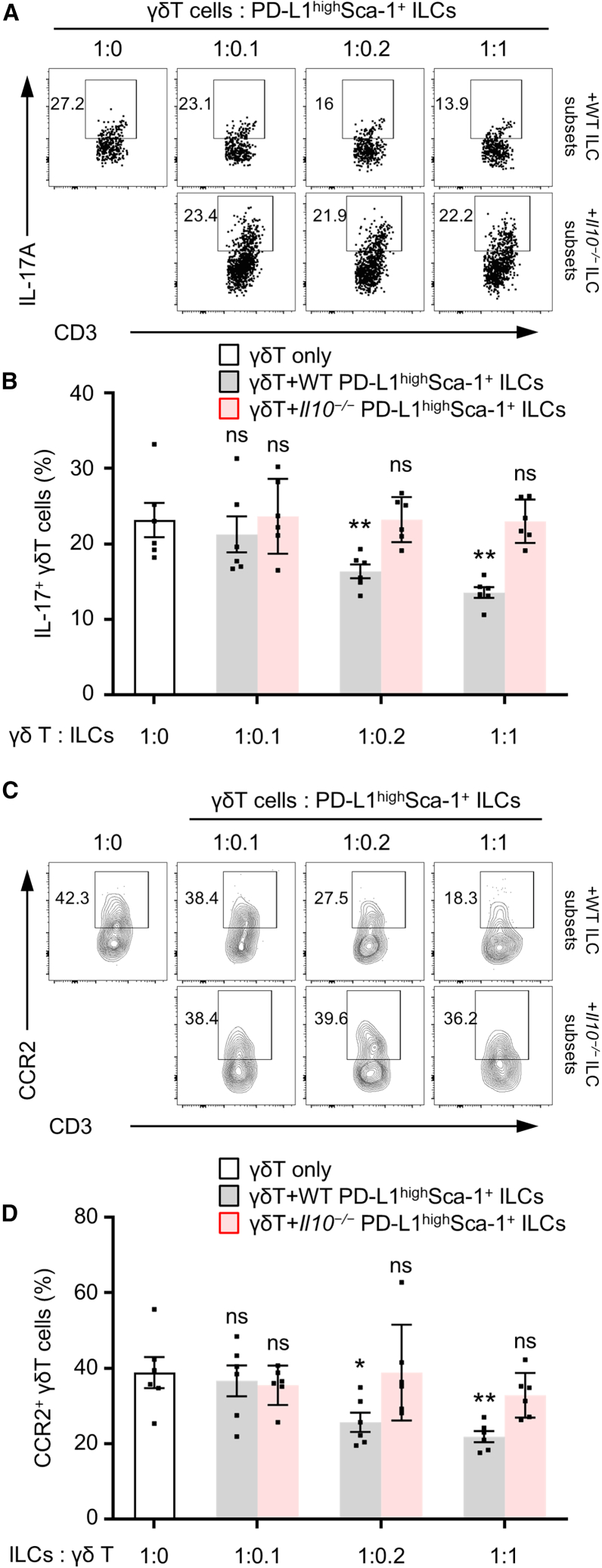
PD-L1^hi^Sca-1^+^ ILCs directly suppress IL-17A production and CCR2 expression in γδ T cells via IL-10 (A) Representative flow cytometry plots of IL-17A expression gated on CD3^+^γδTCR^+^ T cells after 24 h co-culture with WT PD-L1^hi^Sca-1^+^ ILCs (top row) or *Il10*^−/−^ PD-L1^hi^Sca-1^+^ ILCs (bottom row) at γδ T cell:ILC ratios of 1:0, 1:0.1, 1:0.2, and 1:1. (B) Bar graphs quantify the percentage of IL-17A^+^ cells among CD3^+^γδTCR^+^ T cells under each co-culture condition; only WT PD-L1^hi^Sca-1^+^ ILCs suppress IL-17A production in a ratio-dependent manner (*n* = 6). (C) Representative flow cytometry plots of CCR2 expression gated on CD3^+^γδTCR^+^ T cells following 24 h co-culture with WT or *Il10*^−/−^ PD-L1^hi^Sca-1^+^ ILCs at γδ T cell:ILC ratios of 1:0, 1:0.1, 1:0.2, and 1:1. (D) Bar graphs show the percentage of CCR2^+^ cells among CD3^+^γδTCR^+^ T cells under each co-culture condition; WT PD-L1^hi^Sca-1^+^ ILCs downregulate CCR2 in a ratio-dependent manner, whereas *Il10*^−/−^ PD-L1^hi^Sca-1^+^ ILCs do not (*n* = 6). Data are presented as mean ± standard error of the mean (SEM). Statistical significance is indicated as ∗*p* < 0.05, ∗∗*p* < 0.01; ns, not significant. In (B and D), pre-specified pairwise comparisons between γδ T cells co-cultured with WT versus *Il10*^−/−^ PD-L1^hi^Sca-1^+^ ILCs at each γδ T:ILC ratio were analyzed using two-sided unpaired Student’s t-tests; γδ T-only controls were included as reference without formal statistical testing.

We next tested whether the *in vivo* phenotype could be reproduced *in vitro*. γδ T cells were isolated from the draining iLNs of IMQ-treated mice and cocultured for 24 h with ILC2_10_ cells purified from IL-33-primed WT or *Il10*^−/−^ mice at different ratios (ILC2_10_: γδT = 0.1 : 1, 0.2 : 1, and 1 : 1) (Figures 7A–7D).

Coculture with WT ILC2_10_ cells reduced the proportion of IL-17A^+^ γδ T cells in a ratio-dependent manner, whereas coculture with *Il10*^−/−^ ILC2_10_ did not significantly change the frequency of IL-17A^+^ cells (Figures 7A and 7B). CCR2 expression followed a similar trend, as WT ILC2_10_ cells reduced the proportion of CCR2^+^ γδ T cells in a ratio-dependent manner, whereas *Il10*^−/−^ ILC2_10_ cells did not elicit such suppression (Figures 7C and 7D).

## Discussion

We found that in IMQ-induced psoriatic inflammation, an IL-10^+^PD-L1^hi^Sca-1^+^ ILC2 regulatory subset expands in lesional skin and draining lymph nodes (Figures 1E and 1F and 2A–F). Adoptive transfer experiments further revealed that this subset is associated with reduced cutaneous inflammation and decreased γδT17 responses (Figures 4A–4H). Intrinsic IL-10-mediated regulatory circuits^26^^,^^27^^,^^28^ that might complement blockade of the pathogenic IL-23/IL-17 axis in psoriasis have not been clearly defined.^29^^,^^30^ Our data identify lesion-induced ILC2_10_ cells as a candidate cellular component of this pathway, as this subset limits the activity of the effector γδ T17-cell axis *in vivo* (Figures 4D–4H). Building on prior work in CHS,^15^ we further showed that this regulatory activity extends to the γδ T17–CCR2 axis in the IMQ-induced psoriasis-like inflammation model, accompanied by the attenuation of CCR2-related trafficking signals and the local CCL2 milieu (Figures 6A–6C). These effects are IL-10 dependent both *in vivo* and in coculture (Figures 5A–5D and 7A–7D).

Reanalysis of human skin scRNA-seq data (GSE228421)^19^ provides supportive reference evidence that a similar regulatory axis may be detectable in human psoriasis (Figures 3A–3F). In this cohort, PD-L1^+^ ILCs were increased in lesional skin, and a rare population of ILCs coexpressing PD-L1 and IL-10 was more frequently observed in lesions than in nonlesional skin (Figures 3E and 3F). Nevertheless, the expansion of PD-L1^+^ ILCs and the lesion-biased distribution of rare PD-L1^+^IL-10^+^ ILCs in human psoriasis are consistent with the possibility that a related regulatory pathway may also operate in human psoriasis (Figures 3E and 3F). Although GSE228421 is not a direct IL-17A antibody dataset, it represents a human psoriasis treatment cohort using the IL-23p19 inhibitor risankizumab. In this cohort, PD-L1^+^ ILCs were more abundant in pretreatment lesional skin and tended to decrease after treatment, whereas PD-L1^+^IL-10^+^ ILCs remained a very rare population with low overall abundance and substantial variability. These observations raise the possibility that therapeutic targeting of the IL-23/IL-17 axis may influence the PD-L1^+^ ILC compartment in human psoriasis. However, the present study does not allow us to determine whether IL-17A antibody blockade directly affects the PD-L1^hi^Sca-1^+^ ILC2_10_–IL-10 axis, and this translational implication should therefore be interpreted with caution.

IMQ robustly induces the IL-23/IL-17–γδ T17 axis, which is involved in early psoriatic inflammation,^31^^,^^32^ and the central question addressed here is how this γδ T17-cell-centered pathogenic pathway is controlled by an innate IL-10 circuit. The finding that ILC2_10_ cells concomitantly reduce CCR2 expression and IL-17 programming in γδ T17 cells supports the view that although IMQ does not encompass the full spectrum of chronic psoriasis, it is an appropriate tool for investigating the γδ T17-cell pathogenic axis and its regulation (Figures 4E–4H, 6A, 6B, and 7A–7D). Collectively, these findings support the view that the ILC2_10_–γδT17–CCR2 axis reflects an innate IL-10 regulatory circuit engaged primarily during acute or early IL-23/IL-17–γδ T17-driven inflammation, rather than a mechanism that can be generalized to all features of chronic psoriasis. The observation from human scRNA-seq that PD-L1^+^ ILCs and rare PD-L1^+^IL-10^+^ ILCs are skewed toward lesional skin is consistent with the possibility that a similar regulatory axis may be engaged in human psoriatic lesions.

In psoriasis, γδ T17 cells constitute a key effector population involved in the initiation and amplification of lesions,^33^^,^^34^^,^^35^ and prior work has suggested that during inflammation, these cells acquire a CCR2-centered migratory program, enter CCL2-rich lesional skin,^10^^,^^11^^,^^12^ and thereby exacerbate pathology. In this context, the observation that ILC2_10_-cell transfer decreases CCR2 expression on γδ T cells and simultaneously reduces γδ T17-cell accumulation and IL-17A production in lesional skin suggests that ILC2_10_ cells act as regulators that restrict both the CCR2-dependent recruitment of γδ T17 cells and their effector function (Figures 4E–4H and 6A–6C). Overall, these data are consistent with the targeted attenuation of trafficking-associated programming and IL-17 effector activity at the lesion site rather than generalized immunosuppression.

The dependence of the anti-inflammatory activity of ILC2_10_ cells on IL-10 is a defining mechanistic feature of this subset. *Il10*^−/−^ ILC2_10_ cells failed to suppress lesions *in vivo* and did not reduce CCR2 or IL-17A expression in γδ T cells in coculture, supporting a functional link between IL-10 production by ILC2_10_ cells and their regulatory activity (Figures 5A–5D and 7A–7D). In addition, flow cytometric analysis showed that a fraction of CD3^+^γδTCR^+^ cells expressed IL-10R (CD210) (Figure S7), supporting the possibility that ILC2_10_-derived IL-10 can directly act on γδ T cells. This interpretation is further supported by our purified γδ T cell-ILC2_10_ coculture system, in which WT ILC2_10_ cells, but not *Il10*^−/−^ ILC2_10_ cells, suppressed IL-17A production and CCR2 expression under conditions lacking additional accessory populations. In contrast, the PD-L1^hi^Sca-1^+^ phenotype was used here primarily to define and track ILC2_10_ cells, and the present study was not designed to test PD-L1 checkpoint function (Figures 2A–2F and 3B–3D). Therefore, we do not infer a functional contribution of PD-L1, which will require targeted perturbation experiments in future work. Future studies using models such as *Rag2*^−/−^*Il2rg*^−/−^ mice or *Rorc*^−/−^ mice may further clarify how CCR2-dependent γδT17 responses are regulated in the absence of conventional adaptive lymphocytes, γc-dependent lymphoid compartments, or RORγt-dependent IL-17 programs.

In parallel, under conditions in which the γδ T17 axis was suppressed, ILC2_10_-cell transfer was accompanied by an increased proportion of CD206^+^ macrophages and a shift toward higher *Arg1* expression and lower *Nos2* expression in the macrophage compartment (Figures S5C–S5E). These changes are consistent with the transition of the lesional microenvironment toward a more anti-inflammatory state. However, because macrophage-specific perturbation experiments were not performed, we interpret this macrophage remodeling as a contributory, likely secondary change associated with ILC2_10_-mediated protection rather than as a primary causal mechanism.

In summary, ILC2_10_ cells represent an IL-10^+^ regulatory ILC subset that expands in a lesion-focused manner in IMQ-induced psoriatic inflammation and mitigates disease through the IL-10-dependent suppression of CCR2-mediated γδ T17-cell trafficking and IL-17 programming in the skin. Nevertheless, the consistent expansion of PD-L1^+^ ILCs and the lesion-biased presence of IL-10-coexpressing regulatory subsets in both mouse and human datasets suggest that, in addition to strategies that primarily target the pathogenic IL-23/IL-17 effector axis, lesion-localized intrinsic IL-10 regulatory circuits could complement current approaches to controlling psoriatic inflammation.

### Limitations of this study

This study has several limitations. First, all mechanistic experiments were performed in a single acute IMQ-induced mouse model.^36^^,^^37^ The IMQ model relies on TLR7/8 stimulation to acutely activate the IL-23/IL-17 axis and has been criticized for not fully recapitulating chronic psoriatic lesions or responses to biologic therapies.^38^ Therefore, how this axis is maintained and regulated in chronic human psoriasis remains to be determined.

Second, interpretation of the human data is constrained by technical and design issues. IL-10 transcripts are typically low in abundance in single-cell datasets, so quantitative group comparisons and linkage to protein-level or functional readouts will require additional validation (Figures 3C and 3F). Moreover, the human component of this study is limited to reanalysis of public scRNA-seq datasets and does not include protein-level confirmation^39^^,^^40^^,^^41^ or functional assays using patient-derived tissue^42^^,^^43^; therefore, the role of ILC2_10_ cells in human psoriasis should be interpreted cautiously. In addition, the conservative ILC gating and stringent lineage exclusion used to minimize T/B/NK/myeloid contamination likely underestimated the true frequency of ILCs and IL-10^+^ ILCs, and this potential underestimation should be considered when interpreting the apparent rarity of IL-10^+^ ILCs. Because this dataset did not include healthy non-psoriatic skin, nonlesional psoriatic skin was used as the within-patient internal comparator. This point should be considered when interpreting the human data, as nonlesional psoriatic skin is not molecularly identical to healthy normal skin.

Third, the PD-L1^hi^Sca-1^+^ phenotype was used primarily as a marker to define, enrich, and track ILC2_10_ cells, and the present data do not allow conclusions to be drawn about whether PD-L1 itself contributes functionally to regulation (Figures 2A–2F and 3B–3D). This study was not designed to test PD-L1 checkpoint function, and any functional contribution of PD-L1 will require targeted perturbation experiments in future work.

Finally, although transfer of ILC2_10_ cells was associated with an increased proportion of CD206^+^ macrophages and a shift toward higher *Arg1* expression and lower *Nos2* expression in the macrophage compartment (Figures S5C–S5E), it remains unresolved whether this macrophage reprogramming reflects a direct effect of ILC2_10_ cells or a secondary consequence of weakening the γδ T17 axis. Addressing this question will require dedicated mechanistic follow-up studies. Microscopic evaluation in this study was limited to back skin sections, where IMQ-induced epidermal hyperplasia and inflammatory infiltrates were assessed, whereas draining lymph node changes were assessed primarily by flow cytometric analysis. Although *Il10*^−/−^ ILC2_10_ cells failed to confer protection *in vivo*, we did not perform systemic IL-10 neutralization during WT ILC2_10_ transfer experiments; therefore, the present study does not establish whether anti-IL-10 treatment would fully phenocopy *Il10*^−/−^ ILC2_10_ transfer *in vivo*.

## Resource availability

### Lead contact

Further information and requests for resources and reagents should be directed to and will be fulfilled by lead contact Hyuk Soon Kim (hskimxo@dau.ac.kr).

### Materials availability

This study did not generate new unique reagents.

### Data and code availability

• This article analyzes existing, publicly available human psoriasis skin scRNA-seq data. The accession number for the dataset is GEO: GSE228421.
• The custom scripts used for preprocessing and analysis of the scRNA-seq data have been deposited at GitHub (https://github.com/polishunion63/GSE228421_ILC_IL10_PDL1_pipeline) and are publicly available as of the date of publication.
• Processed summary tables generated from the GSE228421 reanalysis, including ILC subset count summaries, PD-L1 and IL-10 expression summaries, pooled summary tables after nILC ≥100 QC, subtype annotation outputs, and exploratory γδ T cell summary outputs, have been deposited in the public GitHub repository listed above and are publicly available as of the date of publication.
• Flow cytometry data reported in this paper and any additional information required to reanalyze the data are available from the lead contact upon reasonable request.

## Acknowledgments

This research was supported by the National Research Foundation of Korea (NRF) grant funded by the Korea government (grant nos. NRF-2021R1A2B5B03002157, RS-2026-25475201) and Global-Learning & Academic Research Institution for Master’s·PhD students, and Postdocs (LAMP) Program of the National Research Foundation of Korea (NRF) grant funded by the Ministry of Education (grant no. RS-2025-25440216). The graphical abstract was created in BioRender. mingeun, J. (2026) https://BioRender.com/mqsxfqm.

## Author contributions

H.S.K. and W.S.C. conceived and supervised the study. M.G.J. designed the study, performed key experiments, and analyzed data. M.Y.C. contributed to experimental execution and data acquisition. K.Y.M., J.W.P., J.S., and J.-A.L. assisted with experiments, assays, and analytical support. M.G.J., W.S.C., and H.S.K. wrote the manuscript. S.H., G.N., and Y.M.K. provided conceptual input, experimental design advice, and critical discussions. All authors read and approved the final manuscript.

## Declaration of interests

The authors declare no competing interests.

## STAR★Methods

### Key resources table

**Table:** 

REAGENT or RESOURCE	SOURCE	IDENTIFIER
**Antibodies**		

Flow: Anti-mouse B220 (RA3-6B2)	Invitrogen	Cat# 11-0452-82; RRID:AB_465054
Flow: Anti-mouse CD4 (GK1.5)	Invitrogen	Cat# 17-0041-82; RRID:AB_469320
Flow: Anti-mouse CD5 (53–7.3)	Invitrogen	Cat# 11-0051-82; RRID:AB_464908
Flow: Anti-mouse CD11b (M1/70)	Invitrogen	Cat# 53-0112-82; RRID:AB_469901
Flow: Anti-mouse CD11c (N418)	Invitrogen	Cat# 11-0114-82; RRID:AB_464940
Flow: Anti-mouse TCR gamma/delta eBioGL3 (GL-3, GL3)	Invitrogen	Cat# 11-5711-81; RRID:AB_465237
Flow: Anti-mouse CD25 (PC61.5)	Invitrogen	Cat# 17-0251-82; RRID:AB_469366
Flow: Anti-mouse CD49b (DX5)	Invitrogen	Cat# 11-5971-82; RRID:AB_465327
Flow: Anti-mouse CD127 (A7R34)	Invitrogen	Cat# 17-1271-82; RRID:AB_469435
Flow: Anti-mouse FcεRIα (MAR-1)	Invitrogen	Cat# 11-5898-82; RRID:AB_465308
Flow: Anti-mouse F4/80 (BM8)	Invitrogen	Cat# 11-4801-82; RRID:AB_2637191
Flow: Anti-mouse Sca-1 (D7)	Invitrogen	Cat# 45-5981-82; RRID:AB_914372
Flow: Anti-mouse TCR β chain (H57-597)	Invitrogen	Cat# 11-5961-85; RRID:AB_465324
Flow: Anti-mouse TER-119 (TER-119)	Invitrogen	Cat# 25-5921-82; RRID:AB_469661
Flow: Anti-mouse CD3 (17A2)	Invitrogen	Cat# 11-0032-82; RRID:AB_2572431
Flow: Anti-mouse IL-17A (eBio17B7)	Invitrogen	Cat# 17-7177-81; RRID:AB_763580
Flow: Anti-mouse Foxp3 (FJK-16s)	Invitrogen	Cat# 12-5773-82; RRID:AB_465936
Flow: Anti-mouse IL-10 (JES5-16E3)	Invitrogen	Cat# 11-7101-82; RRID:AB_465403
Flow: Anti-mouse IL-10 (JES5-16E3)	Invitrogen	Cat# 12-7108-41; RRID:AB_10717511
Flow: Anti-mouse CD16/32, 93	eBioscience	Cat# 14-0161-82; RRID:AB_467133
Flow: Anti-mouse CD3 (17A2)	BioLegend	Cat# 100204; RRID:AB_312661
Flow: Anti-mouse CD3 (17A2)	BioLegend	Cat# 100220; RRID:AB_1732057
Flow: Anti-mouse CD206 (C068C2)	BioLegend	Cat# 141720; RRID:AB_2562248
Flow: Anti-mouse CCR2 (SA203G11)	BioLegend	Cat# 150628; RRID:AB_2810415
Flow: Anti-mouse CCR6 (29-2L17)	BioLegend	Cat# 129804; RRID:AB_1279137
Flow: Anti-mouse PD-L1 (10F.9G2)	BioLegend	Cat# 124314; RRID:AB_10643573
Flow: Anti-mouse CD127 (A7R34)	BioLegend	Cat# 135012; RRID:AB_1937216
Flow: Anti-mouse CD45 (30-F11)	BioLegend	Cat# 103130; RRID:AB_312973
Flow: Anti-mouse CD49b (DX5)	BioLegend	Cat# 108905; RRID:AB_313412
Flow: Anti-mouse CD5 (53–7.3)	BioLegend	Cat# 100605; RRID:AB_312734
Flow: Anti-mouse/human CD11b (M1/70)	BioLegend	Cat# 101206; RRID:AB_312789
Flow: Anti-mouse CD11c (N418)	BioLegend	Cat# 117306; RRID:AB_313775
Flow: Anti-mouse CD19 (1D3/CD19)	BioLegend	Cat# 152410; RRID:AB_2629839
Flow: Anti-mouse CD127 (A7R34)	BioLegend	Cat# 135012; RRID:AB_1937216
Flow: Anti-mouse CD127 (A7R34)	BioLegend	Cat# 135014; RRID:AB_1937265
Flow: Anti-mouse FcεRIα (MAR-1)	BioLegend	Cat# 134306; RRID:AB_1626108
Flow: Anti-mouse F4/80 (BM8)	BioLegend	Cat# 123107; RRID:AB_893500
Flow: Anti-mouse Gr-1 (RB6-8C5)	BioLegend	Cat# 108406; RRID:AB_313371
Flow: Anti-mouse Sca-1 (D7)	BioLegend	Cat# 108122; RRID:AB_893614
Flow: Anti-mouse TCR β chain (H57-597)	BioLegend	Cat# 109206; RRID:AB_313429
Flow: Anti-mouse TER-119 (TER-119)	BioLegend	Cat# 116205; RRID:AB_313706
Flow: Anti-mouse IL-17A (TC11-18H10)	BioLegend	Cat# 506904; RRID:AB_315464
Flow: Anti-mouse IL-17A (TC11-18H10)	BD	Cat# 559502; RRID:AB_397256
Flow: Anti-mouse CD210 (IL-10R) (1B1.3a)	BioLegend	Cat# 112705; RRID:AB_313518

**Chemicals, peptides, and recombinant proteins**		

PMA	Sigma-Aldrich	Cat# P1585-1 MG
Ionomycin calcium salt	Sigma-Aldrich	Cat# I0634-5 MG
Monensin Solution (1000×)	eBioscience	Cat# 00-4505-51
Brefeldin A solution(1000×)	eBioscience	Cat# 00-4506-51
RPMI 1640 Medium	WELGENE	Cat# LM011-01
Recombinant mouse IL-33	BioLegend	Cat# 580506
4% Paraformaldehyde Solution	Biosesang	Cat# PC2031-100-00
Penicillin-Streptomycin (10,000 U/mL)	Gibco	Cat# 15140122
L-Glutamine (200 mM)	Gibco	Cat# 25030081
Fetal Bovine Serum	WELGENE	Cat# S001-07
Foxp3/Transcription Factor Staining Buffer	Thermofisher	Cat# 00-5523-00
BD FACS™ Permeabilizing Solution 2	BD	Cat# 340973
Permeabilization Buffer (10×)	eBioscience	Cat# 00-8333-56
DPBS	WELGENE	Cat# LB001-02
Papanicolaou’s solution 1a Harris’ hematoxylin solution	Sigma-Aldrich	Cat# 1.09253
Eosin Y Solution	Sigma-Aldrich	Cat# 318906
Histosec™ pastilles (without DMSO)	Sigma-Aldrich	Cat# 1.15161
Xylene	DUKSAN	Cat# 2942
Ethyl Alcohol 99.9%	DUKSAN	Cat# 3463

**Critical commercial assays**		

Zombie NIR™ Fixable Viability Kit	BioLegend	Cat# 423106
Multi Tissue Dissociation Kit 1	Miltenyi Biotec	Cat# 130-110-201
easyTM-spin Total RNA Extraction Kit	iNtRON	Car# 17221
AccuPower® CycleScript™ RT PreMix (dT20) (96 T, 20 μL)	BIONEER	Cat# K-2044

**Experimental models: Organisms/strains**		

Mouse: C57BL/6NCrlOri	Orient Bio	N/A
Mouse: B6.129P2-Il10tm1Cgn/J	The Jackson Laboratory	Cat# 002251

**Oligonucleotides**		

qPCR primers	N/A	Table S1

**Software and algorithms**		

Flowjov.10.6.0	Tree Star	https://www.flowjo.com/
Prism 7	GraphPad	http://www.graphpad.com/
Custom scRNA-seq analysis pipeline	This paper	https://github.com/polishunion63/GSE228421_ILC_IL10_PDL1_pipeline

### Experimental model and study participant details

#### Mice

WT C57BL/6 (B6) mice were purchased from Orient Bio, Inc. (Gyeonggi-do, Korea). B6.129P2-Il10tm1Cgn/J (*Il10*^−/−^, Jax Stock #002251). Female mice aged 8 weeks were used in IMQ-induced psoriasis model experiments, whereas female mice aged 6 weeks were used for experiments involving intraperitoneal IL-33 administration to isolate the PD-L1^hi^Sca-1^+^ ILCs. All the mice were bred and maintained in a pathogen-free facility at Konkuk University (Seoul, Korea). All animal experiments were approved by the Institutional Animal Care and Use Committee (IACUC) of Konkuk University.

#### Induction of psoriasis

The dorsal hair on the back of each mouse was first clipped with an electric shaver and then completely removed using a depilatory cream. All animal procedures were performed in accordance with institutional guidelines and were approved by the Institutional Animal Care and Use Committee (IACUC) of Konkuk University (Approval No. KU24197). Psoriatic inflammation was induced by applying 62.5 mg of 5% imiquimod (IMQ) cream (Aldara, 3M Pharmaceuticals) to the dorsal skin once daily for 4 consecutive days,^44^ essentially as previously described with minor modifications. Clinical skin inflammation was scored every day by grading erythema and scaling on a 0–4 scale (0, no sign; 4, very severe). Dorsal skin thickness was measured daily with electronic calipers,^18^ and changes in thickness were recorded as an additional readout of inflammation. On day 4, mice were euthanized and dorsal skin and draining lymph node tissues were collected for subsequent *in vivo* analyses.

#### Expansion of PD-L1^hi^Sca-1^+^ ILCs by IL-33

To induce PD-L1^hi^Sca-1^+^ ILC expansion, wild-type C57BL/6 mice were intraperitoneally injected with 0.3 μg of recombinant mouse IL-33 (carrier-free; BioLegend) diluted in 100 μL of PBS once daily for 5 consecutive days.^22^ After a 2-day resting period without further treatment, the mice were euthanized on day 7 for tissue sorting or analysis.

#### Adoptive transfer of ILC subsets

Spleens from IL-33–sensitized wild-type or *Il10*^−/−^ mice were collected, and PD-L1^hi^Sca-1^+^ ILC2_10_ and PD-L1^lo/−^Sca-1^-^ ILCs (non-ILC2_10_) were purified by fluorescence-activated cell sorting. A total of 2.5 × 10^4^ cells of each subset were injected via the tail vein into wild-type or *Il10*^−/−^ recipient mice 1 day before the start of IMQ treatment^45^

### Method details

#### Flow cytometry and cell sorting

Single-cell suspensions prepared from spleen, iLN, and back skin were first treated with Zombie NIR Fixable Viability Dye (BioLegend) to discriminate live from dead cells. To prevent nonspecific binding, the cells were pre-incubated for 5 min with an anti-CD16/32 antibody before surface staining. The following antibodies against surface proteins were utilized for flow cytometry and cell sorting: CD3 (17A2), CD45 (30-F11), CD127 (A7R34), PD-L1 (10F.9G2), Sca-1 (D7), CD49b (DX5), CD5 (53–7.3), CD11b (M1/70), CD11c (N418), CD19 (1D3/CD19), FcεRIα (MAR-1), F4/80 (BM8), Gr-1 (RB6-8C5), TCRβ (H57-597), TER-119 (TER-119), CD206 (C068C2), CCR2 (SA203G11), CCR6 (29-2L17), and CD210/IL-10R (1B1.3a), which were obtained from BioLegend or Invitrogen. For intracellular staining, antibodies against IL-10 (JES5-16E3), IL-17A (eBio17B7), and Foxp3 (FJK-16s) were used. The cells were stained with surface markers. For intracellular cytokine staining, cells were stimulated with PMA (50 ng/mL; Sigma‒Aldrich, St. Louis, MO), ionomycin (500 ng/mL; Sigma Aldrich), and brefeldin A (3 μg/mL; eBioscience) (for IL-17A); monensin (2 μM; eBioscience) (for IL-10) for 4 h. The cells were subsequently fixed and permeabilized following the manufacturer’s protocols using BD Biosciences’ fixation buffer and a Permeabilizing Solution 2. For intranuclear transcription factor staining, the Foxp3/Transcription Factor Staining Buffer Set (eBioscience) was used as directed. Flow cytometric data were acquired using a FACSCanto II (BD Biosciences) and analyzed using FlowJo v10 (TreeStar). γδ T cells were stained with CD45, CD3, and γδTCR; for IL-10R analysis, γδ T cells were additionally stained with anti-CD210 (IL-10R) antibody (Figure S7); M2-like macrophages with CD45, CD11b, F4/80, and CD206 (Figure S6). To isolate PD-L1^hi^Sca-1^+^ ILCs (Lin(CD3, B220, TCRβ, CD49b, CD11b, CD11c, Gr-1, FceRIα, CD5, TER-119)^−^CD45^+^CD127^+^PD-L1^hi^Sca-1^+^), PD-L1^lo^Sca-1^-^ ILCs (Lin^−^CD45^+^CD127^+^PD-L1^lo/−^Sca-1^−^) from WT or *Il10*^−/−^ C57BL/6 mice, a single-cell suspension of splenocytes was stained with the indicated antibodies and sorted via FACS fusion (BD Biosciences) with a sorting purity of greater than 95%.

#### Single-cell preparation

Spleens and iLNs were mechanically dissociated by passing through a 70 μm nylon mesh (SPL Life Sciences, Gyeonggi-do, Korea) into cold PBS; splenocytes were subsequently incubated with red blood cell (RBC) lysis buffer at room temperature for 5 min, washed once with PBS, pelleted at 380 × g for 5 min at 4 °C and resuspended in Roswell Park Memorial Institute (RPMI)-1640 medium (Gibco) for staining; dorsal skin samples were subjected to two dissociation protocols: in Protocol A, 0.2–0.3 g tissue was cut into 2 mm pieces and digested in RPMI-1640 containing 0.4 mg/mL Liberase TM (Roche) at 37 °C for 30 min with intermittent mixing, digestion was terminated by adding an equal volume of RPMI-1640, the suspension was filtered through a 70 μm mesh, pelleted at 380 × *g* for 5 min, washed with PBS and resuspended in RPMI-1640; in Protocol B, up to 1 g tissue was cut into 1–2 mm pieces, transferred to a gentleMACS C-Tube (Miltenyi Biotec) containing serum-free RPMI-1640 supplemented with 300 μL Enzyme D, 50 μL Enzyme R and 25 μL Enzyme A (Multi Tissue Dissociation Kit 1, Miltenyi Biotec), dissociated using the “37C_cus_m_skin_2” program on a gentleMACS Octo Dissociator, filtered through a 70 μm SmartStrainer, washed twice with RPMI-1640, pelleted at 380 × *g* for 5 min and resuspended in PBS or RPMI-1640 for downstream analysis.

#### γδ T cell cocultured with ILC subsets

γδ T cells were isolated from iLNs of IMQ-induced psoriasis mice using a TCRγ/δ T cell Isolation Kit (Miltenyi Biotec) according to the manufacturer’s instructions. PD-L1^hi^Sca-1^+^ ILCs from the spleens of IL-33-sensitized wild-type and *Il10*^−/−^ C57BL/6 mice were sorted by FACS to >95% purity. For coculture, 5 × 10^3^ γδ T cells were plated in round-bottom 96-well plates together with PD-L1^hi^Sca-1^+^ ILCs at 5 × 10^3^, 1 × 10^3^, or 0.5 × 10^3^ cells per well. The cells were cultured in RPMI-1640 medium and incubated for 24 h. The expression of IL-17A and CCR2 on γδ T cells was subsequently evaluated by flow cytometry.

#### Real-time PCR

Snap-frozen spleen, iLN, and back skin tissues were stored at −80 °C in liquid nitrogen vapor for up to 48 h prior to processing. Tissues were pulverized in liquid nitrogen using a pre-chilled mortar and pestle, and total RNA was extracted using an easy-spin Total RNA Extraction Kit (iNtRON, Burlington, MA, USA) according to the manufacturer’s instructions. The RNA concentration and purity were determined using a NanoDrop spectrophotometer. cDNA was synthesized using the Tetro cDNA Synthesis Kit (Bioline, London, UK). Real-time PCR was performed using the LightCycler 480 SYBR Green I Master Mix (Roche, Basel Switzerland). The sequences of the mouse PCR primers are listed in Table S1.

#### Histology

Mouse back skin was harvested and fixed in 4% paraformaldehyde in PBS. Following graded ethanol dehydration and paraffin embedding, 5 μm serial sections were cut and stained with hematoxylin & eosin or toluidine blue. Representative images were acquired at 100× magnification.

#### Reanalysis of human psoriasis skin scRNA-seq data

Publicly available human psoriasis skin scRNA-seq data (GSE228421) were reanalyzed to define the ILC compartment. Human skin ILCs were identified using transcript-based lineage exclusion plus a relaxed anchor strategy. Cells expressing T cell markers (*TRAC*, *TRBC1*, *TRBC2*, *TRDC*, *TRGC1*, *TRGC2*, and *CD3D*), NK-cell markers (*EOMES* or *PRF1* and *NKG7*), pDC markers (*FCER1A* and *IL3RA*), myeloid markers (*LST1*, *FCGR3A*, *S100A8*, and *S100A9*), B-cell markers (*MS4A1*, *CD79A*, and *CD79B*), or mast-cell markers (*TPSAB1*, *CPA3*, *HDC*, *MS4A2*, and *FCER1B*) were excluded. Among lineage-excluded cells, *GATA3*, *RORC*, *TBX21*, *KIT*, and *ID2*, or ILC2-associated markers (*IL1RL1*, *KLRG1*, *HPGDS*, and *PTGDR2*), were used as anchor genes. ID2-only positive cells were excluded unless IL7R was coexpressed. Subtype-like annotation was then performed using *GATA3*, *IL1RL1* (ST2), *KLRG1*, *HPGDS*, *PTGDR2*, and *IL7R* for ILC2-like cells, *RORC* for ILC3-like cells, and *TBX21* for ILC1-like cells. In the same dataset, exploratory γδ T cell analysis was performed using a transcript-based definition (*TRDC* > 0, *TRGC1/2* > 0, and *TRAC* = = 0). Processed summary tables generated from this reanalysis are publicly available in the GitHub repository listed in the Data and code availability section, together with the scripts used for preprocessing and analysis.

### Quantification and statistical analysis

Data from *in vitro* experiments are shown as the mean ± standard error (SEM) from three or more independent experiments. All animal experiments were performed using five or more mice per group. Statistical analysis was performed using an unpaired two-tailed Student’s *t* test or the Mann-Whitney U test. One-way analysis of variance (ANOVA) with Tukey’s post-hoc test was performed to compare multiple experimental groups. Statistical analysis (∗*p* < 0.05; ∗∗*p* < 0.01) were performed using Prism version 7.0 (GraphPad, San Diego, CA, USA).
