# Supplementary material for: IL-10-producing innate lymphoid cells restrain γδT17-driven psoriatic inflammation
Source: iScience. 2026 Jul 31;29(8):117026. doi: 10.1016/j.isci.2026.117026 (PMC13453000; doi:10.1016/j.isci.2026.117026)
Supplement: Supplementary file 1 [file mmc1.pdf]

iScience, Volume *29*

Supplemental information

IL-10-producing innate lymphoid cells restrain $\gamma\delta$T17-driven psoriatic inflammation

Min Geun Jo, Min Yeong Choi, Keun Young Min, Jeong Won Park, Juhyun Shin, Ji-Ae Lee, Sunsook Hwang, Geunwoong Noh, Young Mi Kim, Wahn Soo Choi, and Hyuk Soon Kim

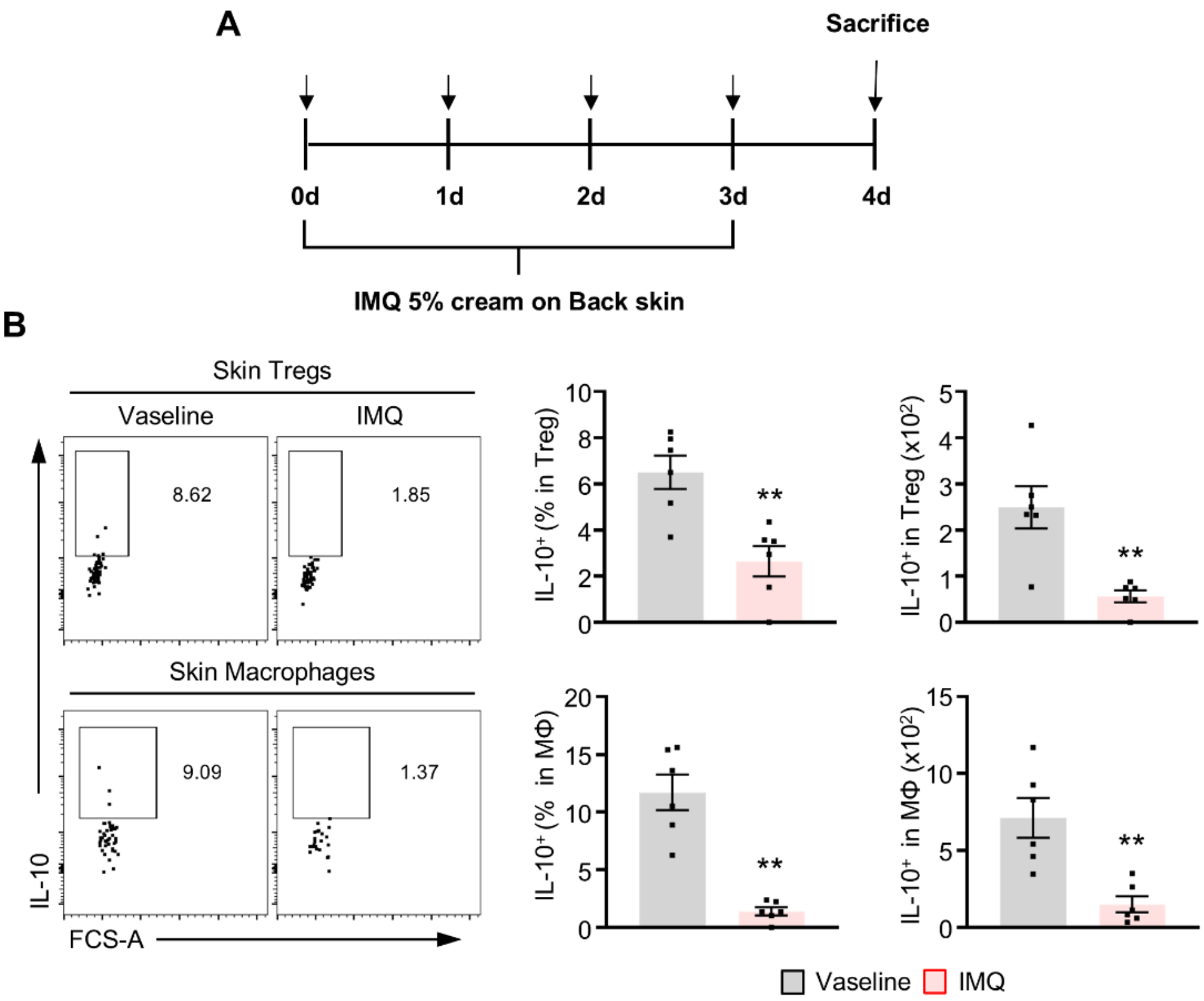

Supplementary Figure 1. IL-10 production by skin Tregs and macrophages is reduced in IMQ-induced psoriatic inflammation

(A) Schematic of the IMQ-induced psoriatic inflammation protocol. Mice received daily topical application of 5% IMQ cream on the dorsal skin for 4 consecutive days and were sacrificed on day 4. (B) Representative flow cytometry plots (left) and quantification (right) of IL-10^+ cells among skin Tregs (top) and skin macrophages (bottom) from Vaseline- and IMQ-treated mice. Bar graphs show the frequency (left) and absolute number (right) of IL-10^+ Tregs or IL-10^+ macrophages per group, demonstrating a marked reduction of IL-10 production in both populations following IMQ treatment. Data are presented as mean ± standard error of the mean (SEM). Statistical significance is indicated as *p < 0.05, **p < 0.01; ns, not significant (two-tailed unpaired Student's t-test). IMQ, imiquimod; Treg, regulatory T cell; Mφ, macrophage.

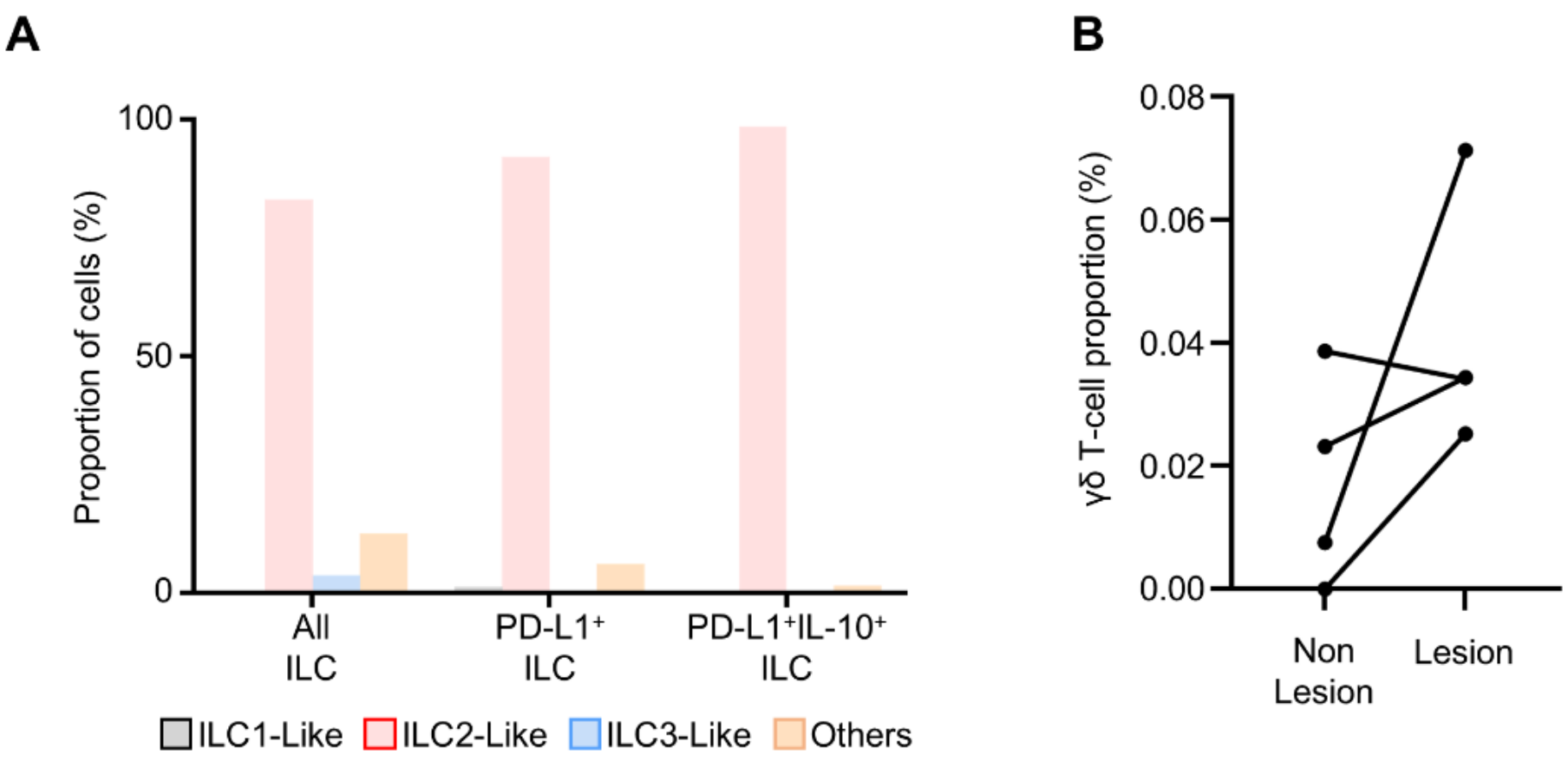

Supplementary Figure 2. Additional analyses of human psoriasis skin scRNA-seq data

(A) Stacked bar plot showing the proportions of ILC2-like, ILC3-like, and ILC1-like cells among all ILCs, PD-L1$^+$ ILCs, and PD-L1$^+$IL-10$^+$ ILCs in the GSE228421 human psoriasis skin scRNA-seq dataset. The predominant fraction of human skin ILCs displayed an ILC2-like signature, and this tendency was further enriched among PD-L1$^+$ ILCs and PD-L1$^+$IL-10$^+$ ILCs. Subtype-like annotation was performed as described in the Methods. (B) Exploratory transcript-based analysis of γδ T cells in the same dataset. Sample-level γδ T-cell proportions in paired V1 non-lesional and lesional samples are shown after exclusion of one non-lesional outlier sample with an extreme value. Because γδ T-cell counts were sparse overall and the analysis was sensitive to sample-level variability, these data are presented as exploratory.

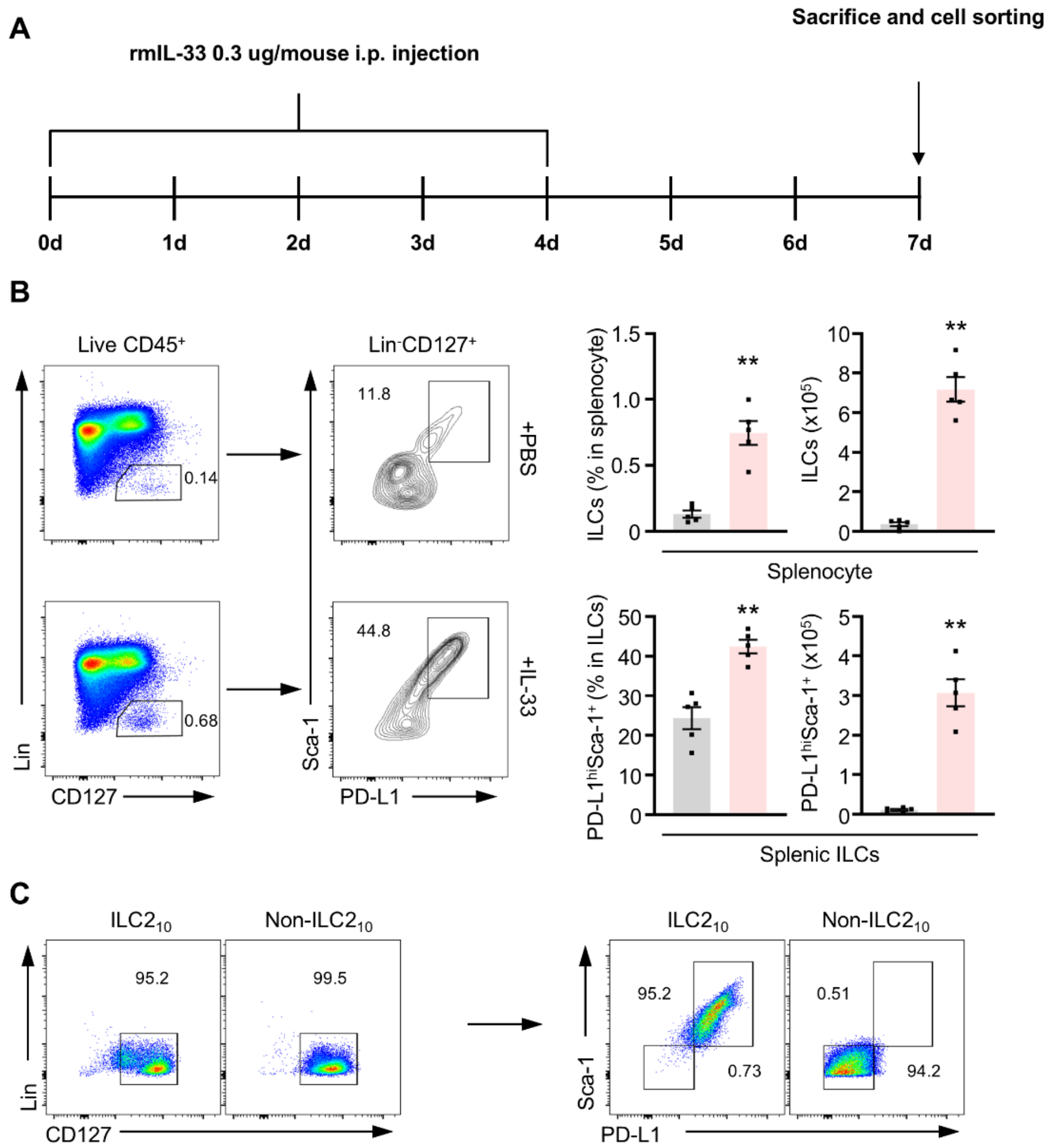

Supplementary Figure 3. Systemic IL-33 administration expands splenic PD-L1^{hi}Sca-1^{+} ILCs

(A) Schematic of the IL-33 treatment regimen. Wild-type mice received daily intraperitoneal injections of recombinant IL-33 (0.3 μg per mouse) for 5 consecutive days, followed by a 2-day rest and tissue collection on day 7 for cell sorting. (B) Representative flow cytometry plots showing splenic Lin^{-}CD127^{+} ILCs and PD-L1 versus Sca-1 expression within the ILC gate from naive and IL-33–treated mice. Bar graphs summarize the frequency of ILCs among splenocytes (top left) and absolute ILC counts (top right), as well as the percentage of PD-L1^{hi}Sca-1^{+} cells within splenic ILCs (bottom left) and absolute PD-L1^{hi}Sca-1^{+} ILC counts (bottom right). IL-33

treatment markedly increases both total splenic ILCs and the PD-L1^{hi}Sca-1^{+} subset. Data are presented as mean ± standard error of the mean (SEM). Statistical significance is indicated as *$p < 0.05$, **$p < 0.01$; ns, not significant (two-tailed unpaired Student’s t-test). IL-33, interleukin-33; ILC, innate lymphoid cell. (C) Representative post-sort flow cytometry plots showing the purity of PD-L1^{hi}Sca-1^{+} ILCs (ILC2$_{10}$) and PD-L1$^{lo/-}$Sca-1^{-} ILCs (non-ILC2$_{10}$) sorted from IL-33–treated spleens, confirming successful enrichment of the two populations used for adoptive transfer experiments.

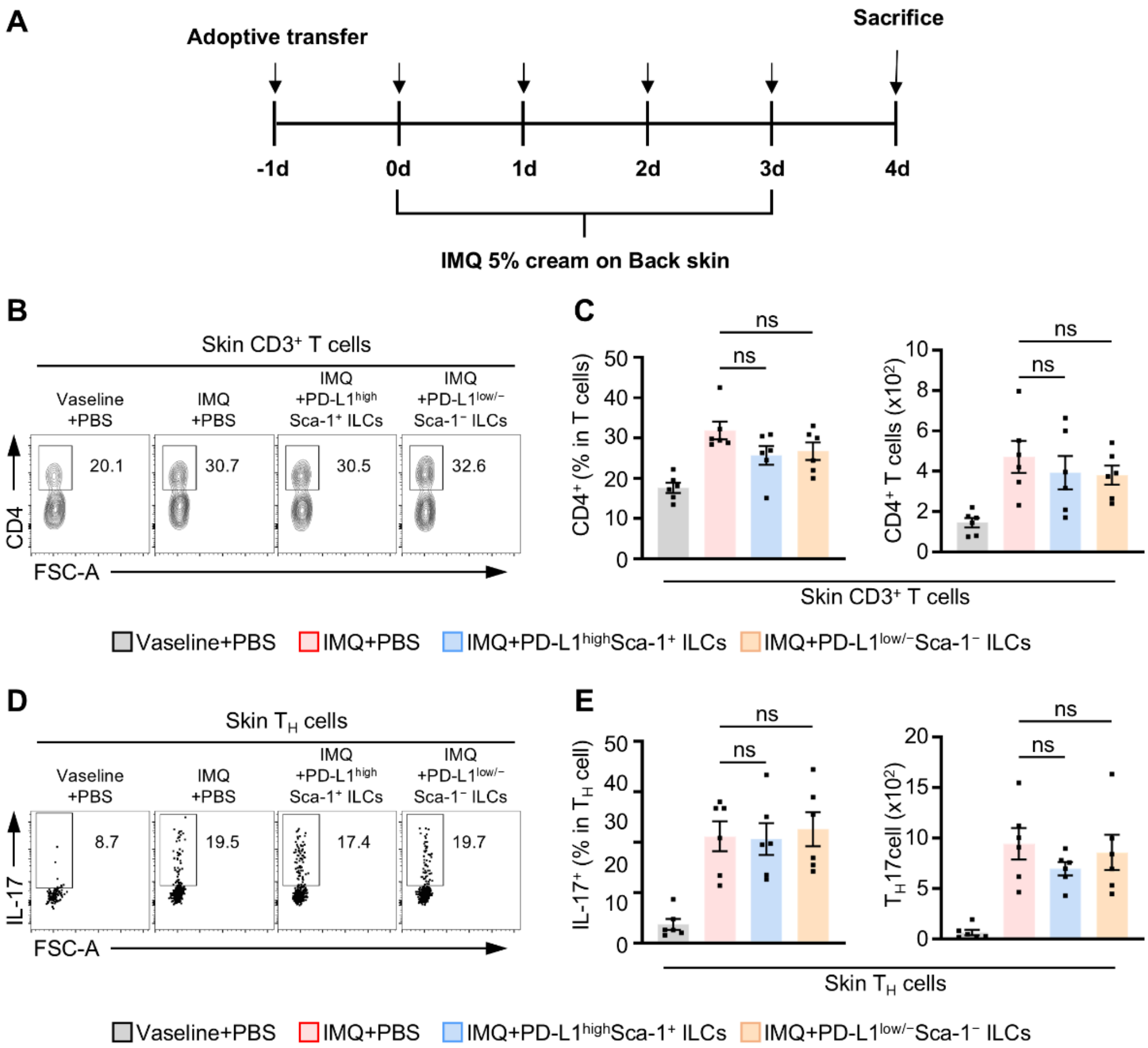

Supplementary Figure 4. PD-L1^{hi}Sca-1^{+} ILC transfer does not significantly alter conventional Th17 responses in psoriatic skin

(A) Schematic of the adoptive transfer experiment: PD-L1^{hi}Sca-1^{+} ILCs or PD-L1$^{lo/-}$Sca-1^{-} ILCs were intravenously transferred 1 day before IMQ application, followed by daily IMQ treatment for 4 days and sacrifice on day 4. (B) Representative FACS plots of skin CD3^{+} T cells (CD4 versus FSC-A) from Vaseline+PBS, IMQ+PBS, IMQ+PD-L1^{hi}Sca-1^{+} ILCs, and IMQ+PD-L1$^{lo/-}$Sca-1^{-} ILCs groups. (C) Bar graphs showing the frequency (left) and absolute number (right) of CD4^{+} T cells among skin CD3^{+} T cells in each group; PD-L1^{hi}Sca-1^{+} ILC transfer does not significantly change total CD4^{+} T cell accumulation. (D) Representative FACS plots of IL-17 expression gated on skin

Th cells ($CD4^+$ T cells) from the same treatment groups. (E) Bar graphs showing the percentage of IL-17^+ cells among skin Th cells (left) and the absolute number of IL-17^+ Th17 cells (right); no significant differences are observed between IMQ+PBS, IMQ+PD-$L1^{hi}$Sca-1^+ ILCs, and IMQ+PD-$L1^{lo/-}$Sca-1^- ILCs groups. Data are presented as mean ± standard error of the mean (SEM). Statistical significance is indicated as $^*p < 0.05$, $^{**}p < 0.01$; ns, not significant (two-tailed unpaired Student's t-test).

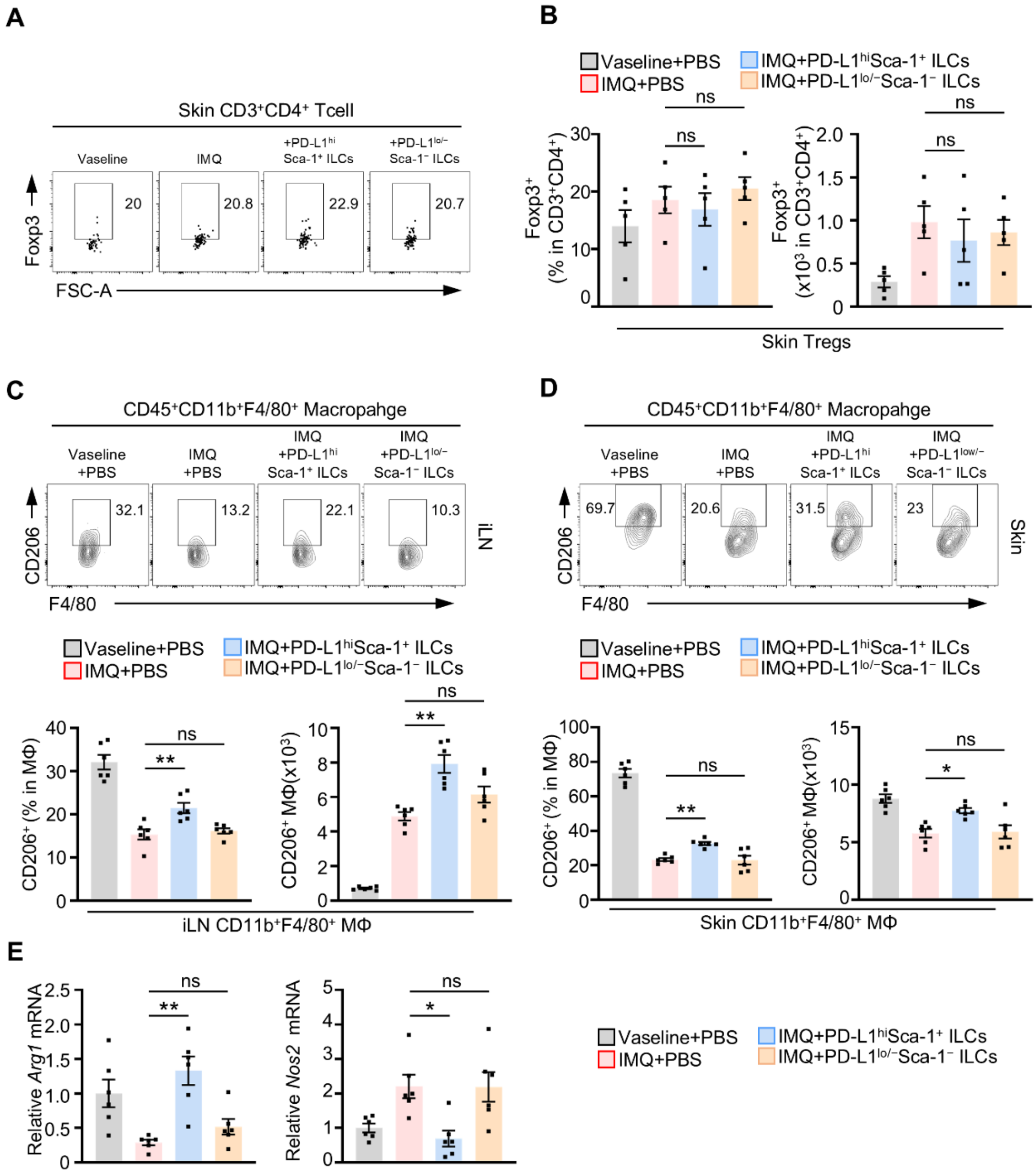

Supplementary Figure 5. Foxp3⁺ Treg and macrophage changes after PD-L1^hiSca-1^+ ILC transfer

(A) Representative FACS plots of Foxp3 expression in skin CD3⁺CD4⁺ T cells from Vaseline+PBS, IMQ+PBS, IMQ+PD-L1^hiSca-1^+ ILC, and IMQ+PD-L1^lo/-Sca-1^- ILC groups. (B) Quantification of the frequency and absolute number of Foxp3⁺ cells among skin CD3⁺CD4⁺ T cells (skin Tregs). Adoptive transfer of PD-L1^hiSca-1^+ ILCs did not significantly alter the frequency or absolute number of skin Foxp3⁺ Tregs. (C)

Representative FACS plots of CD206 expression gated on $CD45^{+}CD11b^{+}F4/80^{+}$ macrophages in iLN. Bar graphs show the percentage and absolute number of $CD206^{+}$ macrophages; PD-$L1^{hi}$Sca-1^{+} ILC transfer increases $CD206^{+}$ iLN macrophages compared with IMQ+PBS. (D) Representative FACS plots and bar graphs of $CD206^{+}$ macrophages in back skin from the same treatment groups; PD-$L1^{hi}$Sca-1^{+} ILCs increase $CD206^{+}$ skin macrophage frequency and counts. (E) qPCR analysis of skin *Arg1* (left) and *Nos2* (right) mRNA normalized to β-actin; PD-$L1^{hi}$Sca-1^{+} ILC transfer upregulates *Arg1* and downregulates *Nos2* compared with IMQ+PBS. Data are presented as mean ± standard error of the mean (SEM). Statistical significance is indicated as *$p < 0.05$, **$p < 0.01$, ns not significant (two-tailed unpaired Student's t-test).

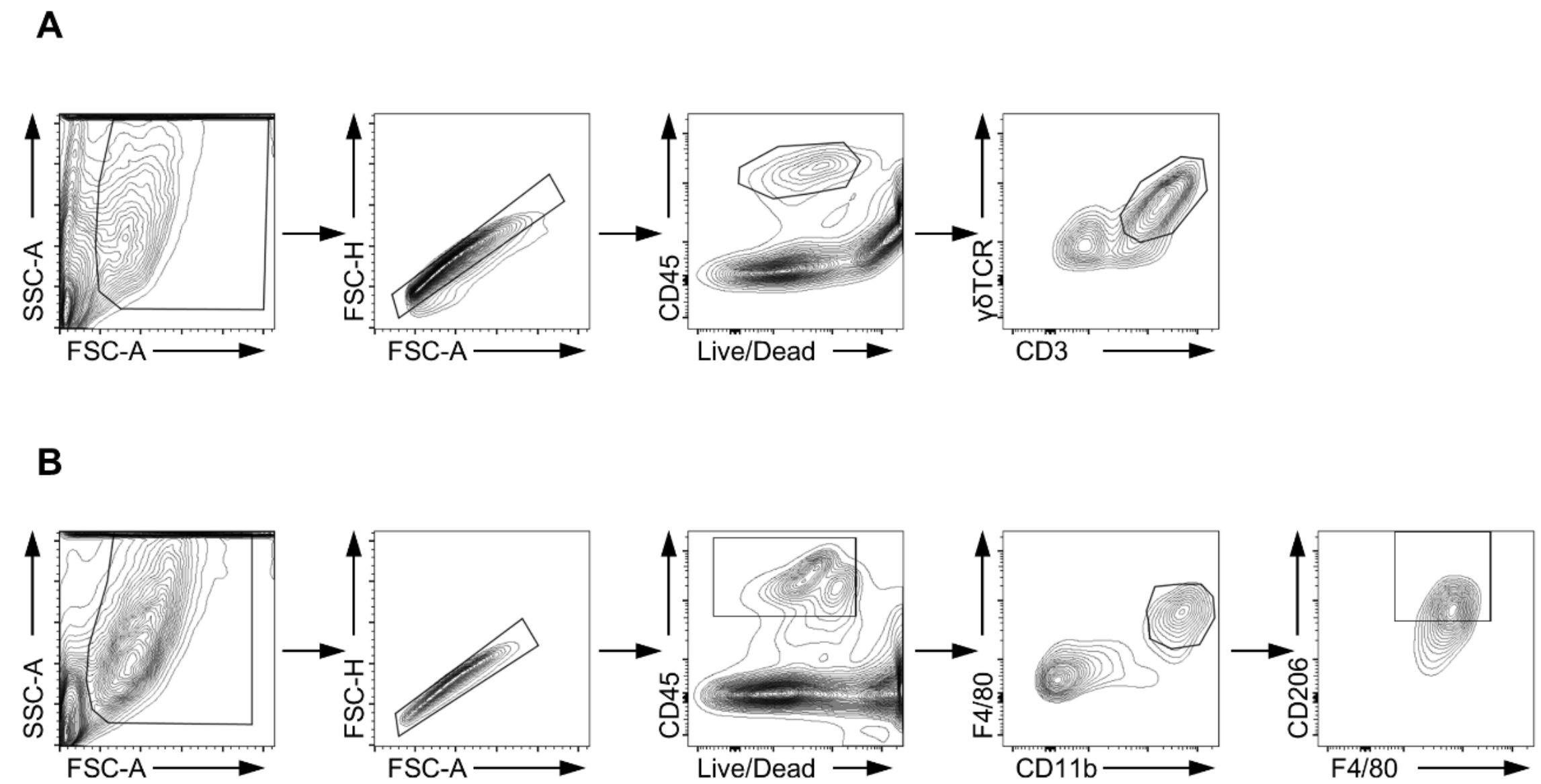

Supplementary Figure 6. Gating strategy for γδ T cells and M2-like macrophages

(A) Representative flow cytometric gating strategy used to identify γδ T cells (live CD45$^+$CD3$^+$γδTCR$^+$). (B) Representative gating strategy used to identify M2-like macrophages (live CD45$^+$CD11b$^+$F4/80$^+$CD206$^+$) in skin and iLN.

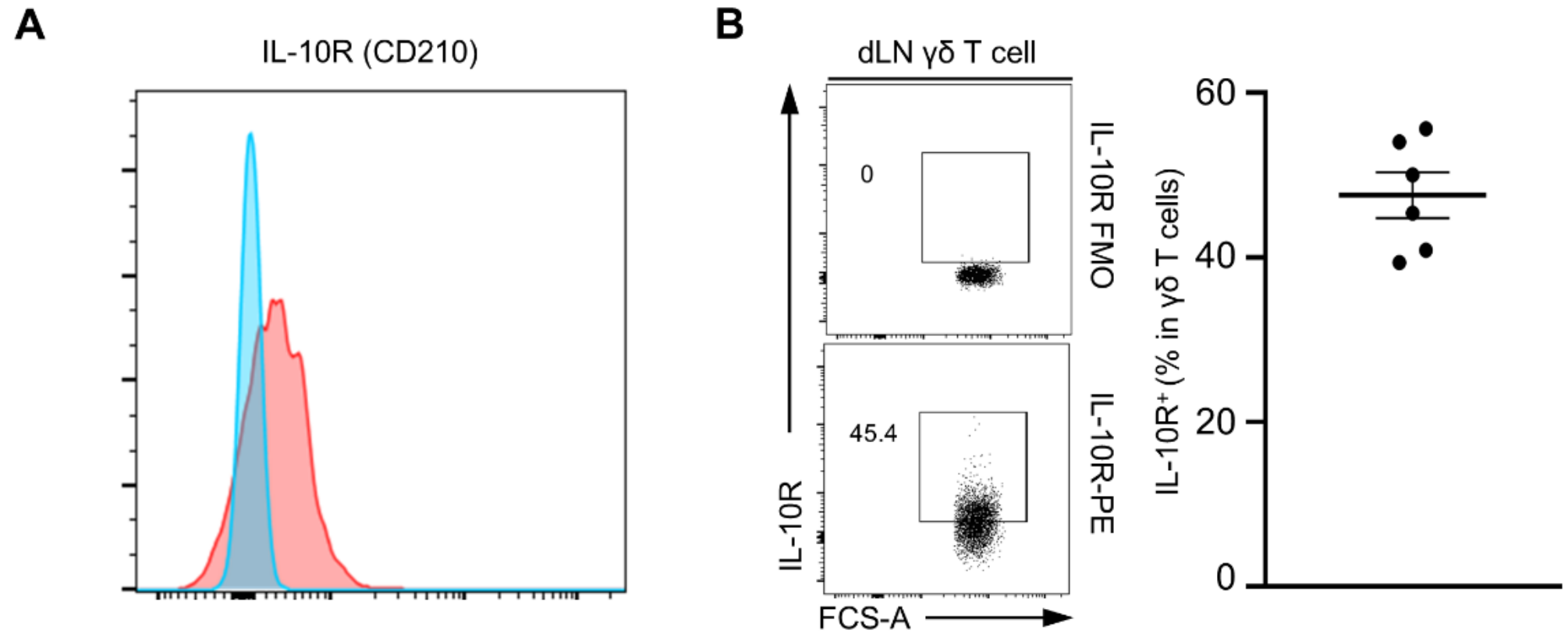

Supplementary Figure 7. A fraction of dLN γδ T cells expresses IL-10R (CD210). (A) Representative overlay histogram showing IL-10R (CD210) staining on gated dLN $CD3^{+}TCR\gamma\delta^{+}$ cells from IMQ-treated mice. Isotype control is shown in blue and anti-CD210 staining is shown in red. (B) Representative flow cytometry plots and quantification of $IL\text{-}10R^{+}$ cells among dLN $CD3^{+}TCR\gamma\delta^{+}$ cells. Each dot represents one mouse.

**RT-qPCR**		
Gene	Forward primer	Reverse primer
Il17a	GGGAGAGCTTCATCTGTGTC	CCTTCACATTCTGGAGGAAGTC
Ccl2	GCTACAAGAGGATCACCAGCAG	GTCTGGACCCATTCCTTCTTGG
Arg1	GGTTTTTGTTGTTGCGGTGTTC	CTGGGATACTGATGGTGGGATGT
Nos2	GAGACAGGGAAGTCTGAAGCAC	CCAGCAGTAGTTGCTCCTCTTC

Supplementary Table 1. List of primer sequences.
